# Skeletal muscle metabolomics and blood biochemistry analysis reveal metabolic changes associated with dietary amino acid supplementation in dairy calves

**DOI:** 10.1038/s41598-018-32241-4

**Published:** 2018-09-14

**Authors:** Kuai Yu, Manolis Matzapetakis, Daniel Valent, Yolanda Saco, André M. De Almeida, Marta Terré, Anna Bassols

**Affiliations:** 1 Departament de Bioquímica i Biologia Molecular, Facultat de Veterinària, Universitat Autònoma de Barcelona Cerdanyola del Vallès, 08193 Barcelona, Spain; 2 Servei de Bioquímica Clínica Veterinària, Facultat de Veterinària, Universitat Autònoma de Barcelona Cerdanyola del Vallès, 08193 Barcelona, Spain; 3 ITQB NOVA, Instituto de Tecnologia Química e Biológica António Xavier, Universidade Nova de Lisboa, Oeiras, Portugal; 4 Instituto Superior de Agronomia, Universidade de Lisboa, Lisbon, Portugal; 5 Departament de Producció de Remugants, Institut de Recerca i Tecnologia Agroalimentàries Caldes de Montbui, 08140 Barcelona, Spain

## Abstract

The effects of different amino acid (AA) supplementations of milk protein-based milk replacers in pre-ruminant calves from 3 days to 7 weeks of age were studied. Animals were divided into 4 groups: Ctrl) Control group fed with milk protein-based milk replacer without supplementation; GP) supplementation with 0.1% glycine and 0.3% proline; FY) supplementation with 0.2% phenylalanine and 0.2% tyrosine; MKT) supplementation with 0.62% lysine, 0.22% methionine and 0.61% threonine. For statistical analysis, *t*-test was used to compare AA-supplemented animals to the Ctrl group. At week 7, body weight and average daily gain (ADG) were measured and blood samples and skeletal muscle biopsies were taken. Blood biochemistry analytes related to energy metabolism were determined and it was shown that MKT group had higher serum creatinine and higher plasma concentration of three supplemented AAs as well as arginine compared with the Ctrl group. GP group had similar glycine/proline plasma concentration compared with the other groups while in FY group only plasma phenylalanine concentration was higher compared with Control. Although the AA supplementations in the GP and FY groups did not affect average daily gain and metabolic health profile from serum, the metabolome analysis from skeletal muscle biopsy revealed several differences between the GP-FY groups and the Ctrl-MKT groups, suggesting a metabolic adaptation especially in GP and FY groups.

## Introduction

Traditionally, bovine milk is considered to be the standard for feeding dairy cattle^1^ and before 1956, whole milk was the main feed for calves^2^. Due to the increasing demand for dairy products, many dairy cattle farms started feeding calves with commercial milk replacers (MRs)^3^. MR quality can vary according to protein source. In milk protein-based MR (MPMR), the protein source originates from milk protein derivatives, and they are considered to have the best quality as they are easier to be digested by young calves^4^. However, due to differences in the levels of certain amino acids (AAs) between MRs and bovine milk, MR AA composition may be inadequate for optimal calf growth, as has been described for infant formulas and human milk^5,6^.

Among the AAs required for pre-ruminant calf’s growth, methionine (M), lysine (K) and threonine (T) are the most studied due to their low concentration in both milk and milk replacers^7–10^. There are indications of improved average daily gain (ADG) and feed efficiency intake in MPMR with MKT supplementation^10^ but other studies suggested no performance benefits comparing MPMR supplemented with methionine^1^, or with threonine and isoleucine^11^.

In humans the comparison of human milk amino acid profile with that of infant formula has been used to propose AA supplementation or ingredient composition in infant formula^5,6^. The comparison of cow milk AA profile^12^ with the skimmed-milk based MR (used as a control in the present study) evidenced than some AAs are deficient in MR compared with cow milk. For instance, proline (P), tyrosine (Y) and phenylalanine (F) were 7.6, 36.9, and 17.5% lower in the control MR in comparison with bovine whole milk^12^. Furthermore, these AAs may be interesting because of their role in the organism, for example proline and glycine (G) are very abundant in collagen^13^, the main structural protein of connective tissue. Phenylalanine and tyrosine play a major role in the neural system as well as the hormonal regulation of metabolism as they are precursors for the synthesis of catecholamines (epinephrine, norepinephrine and dopamine) and thyroid hormones^13^. Moreover, a recent study concludes that phenylalanine and tryptophan could be limiting growth in calves fed following an enhanced-growth feeding program^14^.

Even though many studies described the growth effects of AA supplementation, the effect on tissue composition has rarely been addressed, particularly from the metabolomics perspective. The hypothesis of the present work is that inclusion of these AAs would be able to modify the metabolome of the skeletal muscle since they affect growth, are important components of collagen or are precursors of anabolic or catabolic hormones. The metabolome, the complete set of small-molecules found within a biological sample, is directly related to the metabolic activity, protein activity and/or gene expression^15,16^. The skeletal muscle is suitable for metabolomic approaches, as several studies have been applied not only in differentiating meat characters of different cattle breeds^17^, geographic origins^18,19^, post mortem periods^20^ and sheep breeds and farming conditions^21^, but also in evaluating meat conservation and aging through meat exudate^22^.

The objective of this study is to determine the effects of a MPMR-based diet supplemented with three groups of amino acids (FY, GP, MKT) fed to dairy calves from 3 days up to 7 weeks of age on the ADG and blood biochemical analytes. The second goal is, by using the NMR-metabolomic technology, to monitor the impact of AA supplementations of MPMR on the skeletal muscle metabolome, hence asserting their importance for the dairy industry.

## Results

### Performance data

Initial and biopsy time body weight and ages were measured for the four groups in the study, as shown in Table 1. No differences were observed between groups, although a statistical tendency for higher ADG was observed in MKT group compared with Ctrl (*p* = 0.096) and FY groups (*p* = 0.097).

**Table 1:** Performance data (Mean ± SE) in calves fed with different AA supplementations.

Group	Initial BW (kg)	BW at biopsy (kg)	Initial age (days)	Age at biopsy (days)	ADG (g/d)
Ctrl	44.2 ± 1.66	77.8 ± 1.73	3.3 ± 0.77	48.5 ± 1.04	740 ± 17.5
GP	47.2 ± 1.74	81.9 ± 2.46	2.4 ± 0.26	48.1 ± 0.83	759 ± 23.6
FY	42.3 ± 2.40	75.6 ± 2.58	2.6 ± 0.38	47.5 ± 0.96	740 ± 17.9
MKT	44.6 ± 2.70	80.8 ± 2.75	2.4 ± 0.65	47.6 ± 0.94	798 ± 26.9

Ctrl: Control milk replacer; GP: Milk replacer supplemented with glycine and proline; FY: Milk replacer supplemented with phenylalanine and tyrosine and MKT: Milk replacer supplemented with lysine, methionine and threonine (n = 8 per group). ADG = Average daily gain; BW = Body weight. Results were analysed by *t*-test for comparisons between Ctrl and treatment groups.

### Blood profile and multivariate analysis of data

At the end of the treatment (week 7), several serum biochemical analytes related to health and nutritional status as well as plasma free AA profile were determined (Table 2).

**Table 2:** Serum biochemical analytes and plasma AA concentrations in calves fed with different AA supplementations.

Concentration (Mean ± SE)	Ctrl	GP	FY	MKT
**Serum biochemical profile**				
ALT (U/L)	10.4 ± 0.7	11.1 ± 1.5	9.2 ± 0.5	10.2 ± 1.2
AST (U/L)	37.5 ± 3.2	39.6 ± 5.7	33.5 ± 4.9	42.2 ± 5.9
Cholesterol (mg/dL)	64.9 ± 8.2	65.2 ± 6.5	68.0 ± 12.7	72.7 ± 6.6
Creatinine (mg/dL)	0.60 ± 0.04^a^	0.68 ± 0.05^a^	0.57 ± 0.03^a^	0.71 ± 0.02^b^
GGT (U/L)	18.5 ± 2.0	14.2 ± 1.1	16.6 ± 1.5	17.9 ± 0.6
Glucose (mg/dL)	86.3 ± 2.8	92.2 ± 4.3	96.9 ± 6.1	92.8 ± 5.4
Haptoglobin (mg/mL)	0.12 ± 0.01	0.13 ± 0.01	0.18 ± 0.06	0.16 ± 0.04
IGF-I (ng/mL)	264.4 ± 35.9	282.2 ± 31.6	245.9 ± 28.9	318.5 ± 33.3
Insulin (μg/L)	0.75 ± 0.13	1.04 ± 0.20	0.85 ± 0.11	1.07 ± 0.22
NEFAs (mM)	0.15 ± 0.03	0.16 ± 0.03	0.13 ± 0.02	0.15 ± 0.02
TGs (mg/dL)	19.91 ± 5.23	20.99 ± 4.59	16.20 ± 1.87	23.51 ± 5.37
TP (g/dL)	4.40 ± 0.11	4.42 ± 0.12	4.57 ± 0.15	4.75 ± 0.13
Urea (mg/dL)	10.54 ± 0.65	13.40 ± 1.44	9.84 ± 0.96	11.63 ± 1.07
**Plasma AAs profile**				
Alanine (μM)	236.9 ± 16.6	228.9 ± 18.1	230.8 ± 30.8	259.6 ± 13.9
Arginine (μM)	223.7 ± 17.4^a^	264.2 ± 23.1^a^	236.7 ± 23.6^a^	291.9 ± 18.7^b^
Asparagine/Serine (μM)	177.9 ± 14.0	195.4 ± 18.9	189.2 ± 23.8	190.6 ± 10.3
Cysteine (μM)	39.0 ± 5.5	38.3 ± 5.9	38.9 ± 6.78	40.4 ± 5.9
Glutamic acid (μM)	77.8 ± 5.2	68.0 ± 6.8	65.2 ± 5.5	75.1 ± 6.5
Glycine (μM)	326.8 ± 26.1	372.1 ± 25.2	340.6 ± 19.9	368.3 ± 15.3
Histidine/Glutamine (μM)	487.1 ± 30.8	504.0 ± 34.8	485.7 ± 49.1	503.9 ± 24.6
Isoleucine (μM)	137.0 ± 11.0	130.7 ± 12.7	134.7 ± 15.8	129.56 ± 6.9
Leucine (μM)	195.20 ± 19.64	181.37 ± 21.66	188.57 ± 28.64	172.05 ± 8.31
Lysine (μM)	220.3 ± 29.6^a^	231.4 ± 24.3^a^	240.8 ± 43.4^a^	304.3 ± 18.8^b^
Methionine (μM)	36.4 ± 6.1^a^	35.4 ± 6.6^a^	36.1 ± 7.4^a^	73.6 ± 5.1^b^
Phenylalanine (μM)	56.4 ± 6.5^a^	57.5 ± 6.5^a^	79.0 ± 7.7^b^	52.2 ± 3.7^a^
Proline(μM)	137.4 ± 11.3	163.7 ± 15.0	141.3 ± 23.2	147.3 ± 10.9
Threonine (μM)	270.9 ± 10.9^a^	276.6 ± 24.3^a^	270.3 ± 25.9^a^	386.7 ± 26.7^b^
Tryptophan (μM)	49.7 ± 4.4	56.0 ± 6.3	52.2 ± 8.2	54.8 ± 3.6
Tyrosine (μM)	66.7 ± 10.3	69.6 ± 13.8	86.8 ± 8.6	67.2 ± 7.0
Valine (μM)	280.7 ± 18.7	273.4 ± 26.5	272.1 ± 35.3	267.5 ± 10.2

Key: Ctrl: Control milk replacer; GP: Milk replacer supplemented with glycine and proline; FY: Milk replacer supplemented with phenylalanine and tyrosine and MKT: Milk replacer supplemented with lysine, methionine and threonine (n = 8 per group). ALT = alanine aminotransferase; AST = aspartate aminotransferase; GGT = gamma glutamyltransferase; IGF-I = insulin-like growth factor 1; NEFAs = non-esterified fatty acids; TGs = triglycerides; TP = total protein. Results were analysed by *t*-test for comparisons between Ctrl and treatment groups. Values in a row with different superscripts are significant different (*P* < 0.05).

For serum biochemistry analytes, there were no differences between Ctrl and the GP or FY group, whereas MKT group presented higher values of serum creatinine compared to the Ctrl group (*p* = 0.023) as well as a tendency for higher total protein (*p* = 0.059). As for the plasma AA concentration, the three supplemented AAs were higher in the MKT group than in the Ctrl group, as well as the concentration of plasma arginine. In the case of FY group, the concentration is significantly higher for phenylalanine supplemented compared with Ctrl group. In the case of GP, there were no significant differences compared to the Ctrl group.

**Figure 1:**
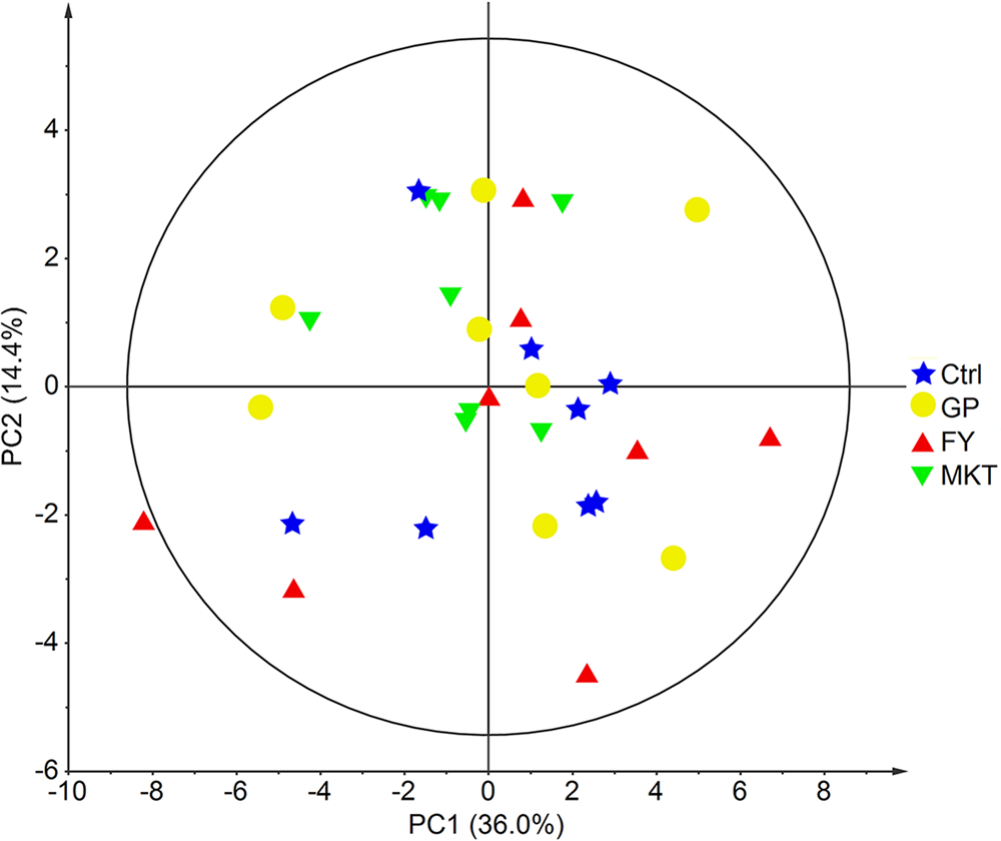
PCA of combined serum biochemical analytes and plasma AAs. Data from dairy calves fed with control milk replacer (Ctrl); milk replacer supplemented with glycine and proline (GP); milk replacer supplemented with phenylalanine and tyrosine (FY) and milk replacer supplemented with lysine, methionine and threonine (MKT) (n = 8 per group). Ellipse Hotelling’s T2 (95%).

Multivariate analysis of the combined data from serum biochemistry and plasma AA using PCA agreed with the univariate results since the different treatment groups did not show major differences (Fig. 1). Pairwise PLS-DA could only discriminate between the Ctrl and the MKT treatment (NC = 2, R^2^Y = 0.91, Q^2^ = 0.60). The VIP variables with values higher than 1.5 and therefore most responsible for the difference were, in decreasing VIP order, methionine, threonine, arginine, creatinine and lysine.

### Muscle tissue metabolomics profile

#### NMR spectra analysis and compound identification

**Figure 2:**
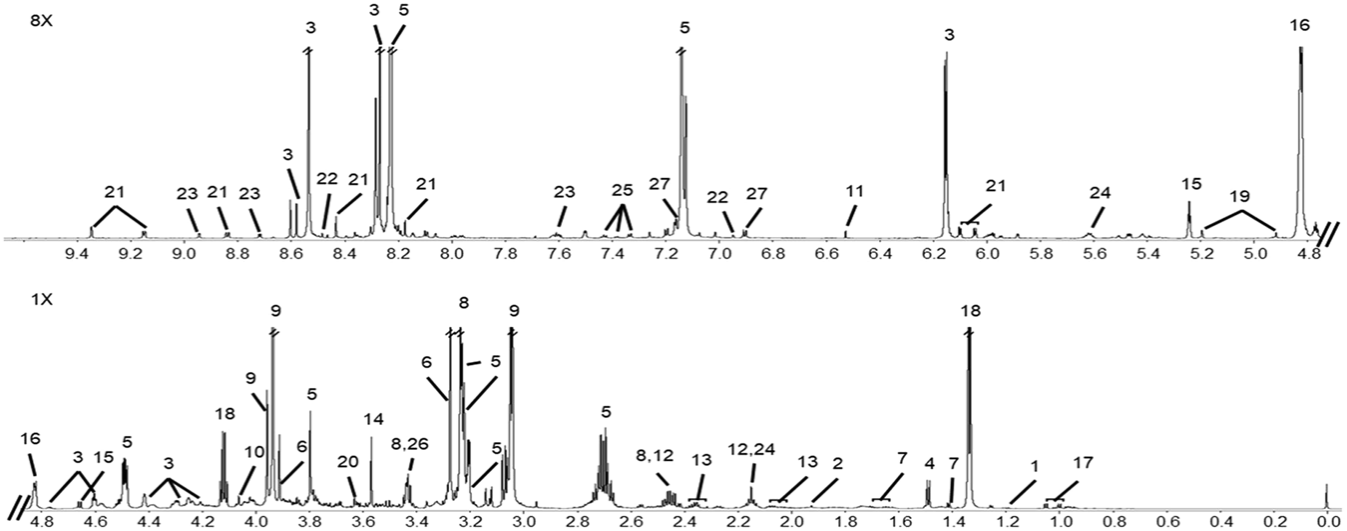
Representative 800 MHz 1H-NMR spectrum of calf muscle aqueous fraction. 1X zoom from 0.0–4.8 ppm and 8X zoom from 4.8–9.4 ppm. Assignments of the 36 metabolites that were quantified: 1: 3-Hydroxybutyrate; 2: Acetate: 3: ADP/AMP/ATP; 4: Alanine; 5: Anserine/Carnosine; 6:Betaine; 7: Cadaverine/Lysine; 8: Carnitine; 9: Creatine/Creatine Phosphate; 10: Creatinine; 11: Fumarate; 12: Glutamine; 13: Glutamate; 14: Glycine; 15: Glucose/Glucose-6-Phosphate; 16: Residual H_2_O (not quantified); 17: Isoleucine/Leucine/Valine; 18: Lactate; 19: Mannose; 20: Myo-Inositol; 21: NAD^+^/NADP^+^; 22: NADH/NADPH; 23: Nicotinurate; 24: O-Acetylcarnitine; 25: Phenylalanine; 26: Taurine; 27: Tyrosine.

Prior to the metabolomics analysis of the muscle tissue extracts, the assignment of the features of the NMR spectrum to specific compounds was made. Selected spectra were assigned using database assisted spectral deconvolution with the Chenomx software platform using its internal database as well as the human metabolite database data (http://www.hmdb.ca/). 2D TOCSY spectra and literature reports^21,22^ were also used to verify the identity of other metabolites. A total of 70 compounds could be successfully identified (Supplementary Table S1). From these, a subset of 36 metabolites was selected for quantification using Chenomx. The selection of this group was made based on the ability to reliably quantify them, their relevance from chemometric multivariate analysis (see chemometric section) and their relevance to the biochemical analytes of this study. The assignment of the selected metabolites on a representative spectrum is shown in Fig. 2.

#### Univariate and multivariate analyses

Upon inspection, two samples (one from GP and one from FY) were found to have very low concentrations. This implies a putative extraction problem and were discarded from the analysis. A total of 30 samples remained and were subsequently used. The mean concentrations of the 36 quantified metabolites corrected for tissue mass are shown in Table 3. According to the *p* values from the *t*-test, no differences between Ctrl and the MKT group could be identified, whereas the GP group had higher carnosine and nicotinurate levels than the Ctrl group. In the FY group, a lower concentration of cadaverine and higher concentration of nicotinurate were found.

**Table 3:** Mean concentrations of quantified metabolites from semitendinosus muscle of calves fed with different AA supplementations. Key: Ctrl: Control milk replacer (n = 8); GP: Milk replacer supplemented with glycine and proline (n = 7); FY: Milk replacer supplemented with phenylalanine and tyrosine (n = 7) and MKT: Milk replacer supplemented with lysine, methionine and threonine (n = 8). Results were analysed by *t*-test for comparisons between Ctrl and treatment groups. Values in a row with different superscripts are significant different (*P* < 0.05).

Metabolites (μMol/100 g muscle ± SE)	Ctrl	GP	FY	MKT
3-Hydroxybutyrate	0.27 ± 0.03	0.29 ± 0.04	0.35 ± 0.03	0.26 ± 0.03
Acetate	0.89 ± 0.11	0.86 ± 0.06	0.90 ± 0.05	0.83 ± 0.08
ADP+ATP+AMP	30.59 ± 3.72	33.92 ± 2.25	29.45 ± 6.25	26.93 ± 4.60
Alanine	10.88 ± 1.32	10.45 ± 1.37	10.17 ± 1.18	10.76 ± 1.06
Anserine	19.36 ± 1.68	22.72 ± 2.30	20.82 ± 2.12	18.99 ± 2.55
Aspartate	0.76 ± 0.08	0.64 ± 0.10	0.85 ± 0.10	0.78 ± 0.12
Betaine	26.61 ± 2.83	29.65 ± 2.63	27.63 ± 2.10	21.04 ± 3.81
Cadaverine	2.01 ± 0.27^a^	1.72 ± 0.30^a^	0.97 ± 0.15^b^	3.10 ± 0.70^a^
Carnitine	27.52 ± 1.62	32.75 ± 2.83	30.42 ± 1.99	24.84 ± 2.24
Carnosine	159.96 ± 7.79^a^	185.24 ± 8.17^b^	190.04 ± 14.08^a^	140.55 ± 15.61^a^
Creatine	281.90 ± 27.39	317.50 ± 24.28	293.29 ± 24.86	254.32 ± 30.07
Creatine phosphate	74.41 ± 14.75	62.08 ± 20.60	83.93 ± 17.42	65.62 ± 14.77
Creatinine	11.48 ± 2.31	10.20 ± 3.08	12.67 ± 2.02	12.21 ± 2.55
Fumarate	0.36 ± 0.08	0.45 ± 0.10	0.27 ± 0.05	0.33 ± 0.06
Glucose	11.06 ± 2.78	12.76 ± 2.37	9.20 ± 1.69	9.25 ± 1.52
Glucose-6-phosphate	12.45 ± 4.90	18.18 ± 4.40	7.52 ± 3.07	6.24 ± 1.72
Glutamate	9.54 ± 0.63	11.08 ± 1.14	9.89 ± 0.72	9.67 ± 1.58
Glycine	24.68 ± 3.23	23.46 ± 3.35	22.73 ± 3.97	22.99 ± 2.95
Isoleucine	1.05 ± 0.13	0.80 ± 0.13	1.01 ± 0.18	0.85 ± 0.08
Lactate	203.66 ± 47.73	300.35 ± 65.52	208.37 ± 61.25	169.21 ± 25.05
Leucine	1.43 ± 0.21	1.19 ± 0.20	1.39 ± 0.28	1.16 ± 0.13
Mannose	2.79 ± 0.64	3.32 ± 0.56	1.66 ± 0.41	1.76 ± 0.23
Myo-inositol	4.82 ± 0.35	4.94 ± 0.52	4.43 ± 0.32	4.56 ± 0.43
NAD^+^	2.63 ± 0.12	2.88 ± 0.19	3.05 ± 0.18	1.94 ± 0.32
NADH	1.00 ± 0.17	0.69 ± 0.10	0.82 ± 0.20	0.79 ± 0.18
NADP	0.10 ± 0.01	0.12 ± 0.01	0.11 ± 0.01	0.11 ± 0.01
Nicotinurate	1.47 ± 0.08^a^	2.14 ± 0.22^b^	1.84 ± 0.07^b^	1.45 ± 0.15^a^
O-Acetylcarnitine	4.57 ± 1.38	3.64 ± 0.53	4.36 ± 0.53	2.95 ± 0.32
Phenylalanine	0.48 ± 0.05	0.42 ± 0.04	0.58 ± 0.07	0.40 ± 0.04
Pyruvate	1.20 ± 0.19	1.40 ± 0.20	1.05 ± 0.11	1.01 ± 0.09
Taurine	19.87 ± 1.34	21.94 ± 2.00	23.03 ± 1.00	18.58 ± 1.96
Tyrosine	0.72 ± 0.08	0.64 ± 0.07	0.89 ± 0.09	0.65 ± 0.08
Tryptophan	0.19 ± 0.02	0.19 ± 0.02	0.17 ± 0.02	0.19 ± 0.02
Valine	2.38 ± 0.27	1.99 ± 0.20	2.18 ± 0.33	1.85 ± 0.20

**Figure 3:**
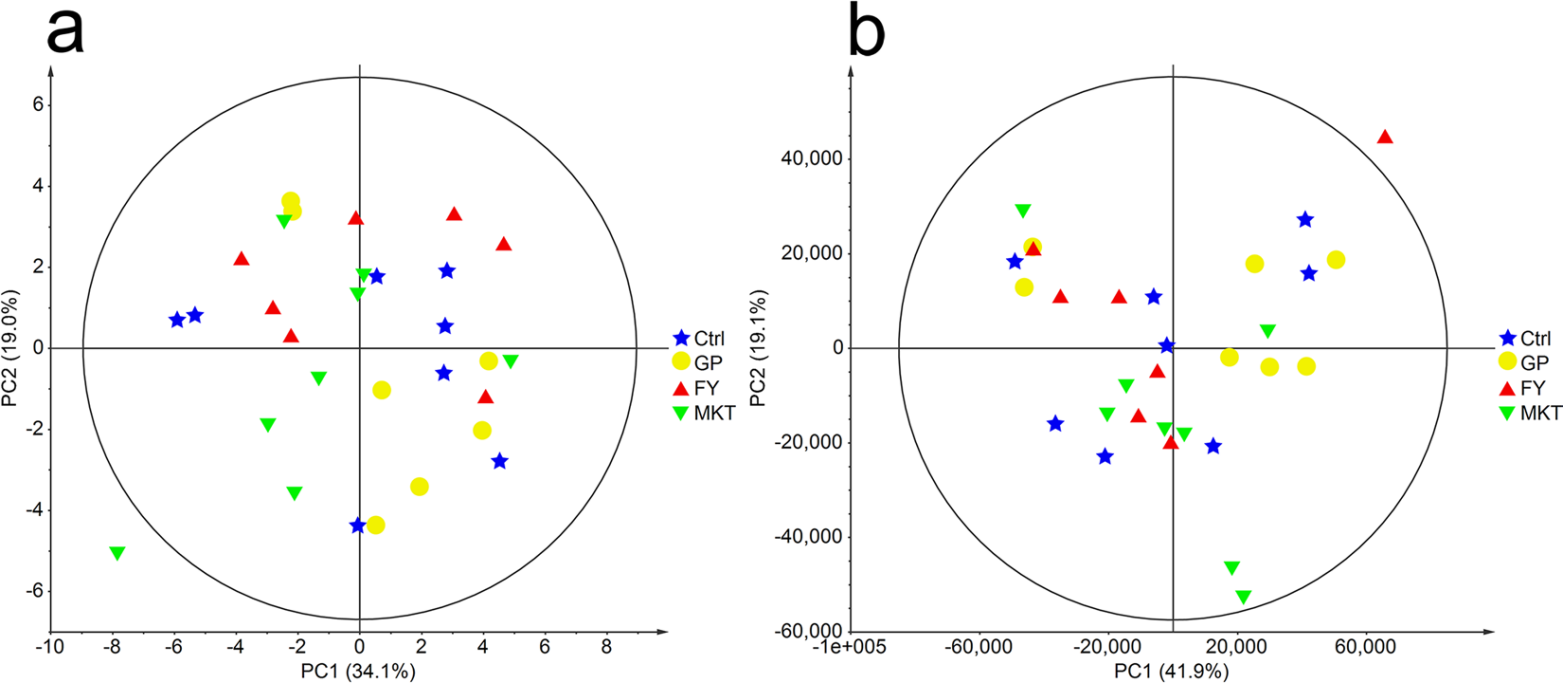
PCAs of **a** Quantified muscle metabolites and **b** Chemometrics data from muscle biopsies. Data from dairy calves fed with control milk replacer (Ctrl, n = 8); milk replacer supplemented with glycine and proline (GP, n = 7); milk replacer supplemented with phenylalanine and tyrosine (FY, n = 7) and milk replacer supplemented with lysine, methionine and threonine (MKT, n = 8). Ellipse Hotelling’s T2 (95%).

Multivariate analyses of the tissue metabolite concentrations, using PCA (Fig. 3a) were consistent with the univariate results. As in the case of the blood profile, the different treatment groups did not show any global differences. Pairwise PLS-DA could only discriminate between the Ctrl-FY groups (NC = 3, R^2^Y = 0.92, Q^2^ = 0.58) and FY-MKT groups (NC = 3, R^2^Y = 0.86, Q^2^ = 0.45). The VIP variables with values higher than 1.4 reveal that the most responsible for the differentiation were, in decreasing VIP order, cadaverine and nicotinurate in Ctrl - FY comparison and cadaverine and NAD^+^ in FY-MKT comparison.

#### Chemometric analysis

To assess if additional regions of the NMR spectra contained information that could be used to discriminate between the experimental groups, multivariate analysis of the full spectra (chemometric) was also employed. As shown in Fig. 3b, no clear separation or clustering could be observed. This is consistent with the multivariate analysis based on the metabolite concentrations. However, PLS-DA could produce a model with acceptable statistical parameter for the comparison of the FY and Ctrl groups (NC = 3, R^2^Y = 1.00, Q^2^ = 0.44). A close look at the VIP data reveals that most compounds with VIP scores over 2.5 are energy-related metabolites, AA, AA derivatives as well as some unassigned compounds (Supplementary Table S2). After a visual inspection of binned spectra, an increase in the total area between 1.422–1.467 ppm and 1.632–1.760 ppm was identified in MKT group (Supplementary Fig. S1). According to the assignment, it was found that these regions mainly contain lysine/cadaverine/arginine/leucine/ornithine as well as some other unassigned metabolites.

#### Comparative analyses of metabolites between AA-treatments

**Figure 4:**
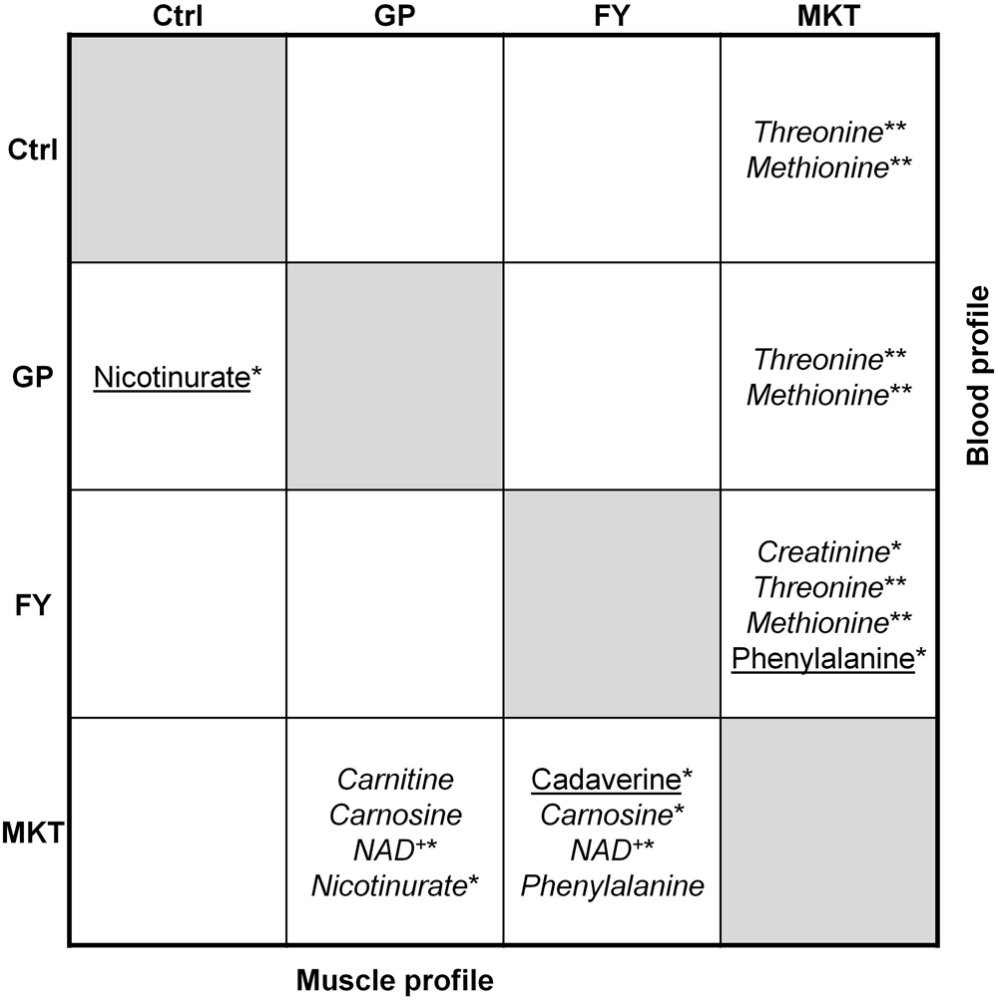
Schematic overview of the differential metabolite profiles in blood (right-up corner) and muscle (down-left corner) between groups. Data from dairy calves fed with control milk replacer (Ctrl); milk replacer supplemented with glycine and proline (GP); milk replacer supplemented with phenylalanine and tyrosine (FY) and milk replacer supplemented with lysine, methionine and threonine (MKT). Blood analytes are shown in the upper-right corner, and muscle metabolites are shown in the down-left corner. Parameters at higher concentration in the row-group vs the column-group are presented in underlined font, whereas parameters at higher concentration in the column-group vs the row-group are presented in italic font. (*P < 0.05, **P < 0.01, no asterisk: statistical tendency 0.05 ≤ *P* ≤ 0.1).

While the focus of this work is on the differences between the treatments and the Ctrl group, the identification of differences among treatments has the potential to provide insights into the processes affected by the modifications in feed composition. Therefore, a one-way ANOVA with Tukey post-hoc test was used to identify the most significant changes among AA groups. The results of this analysis are summarized in the scheme shown in Fig. 4, where the upper portion shows the results for blood and the lower those for muscle tissue (complete information in Supplementary Tables S3 and S4).

**Figure 5:**
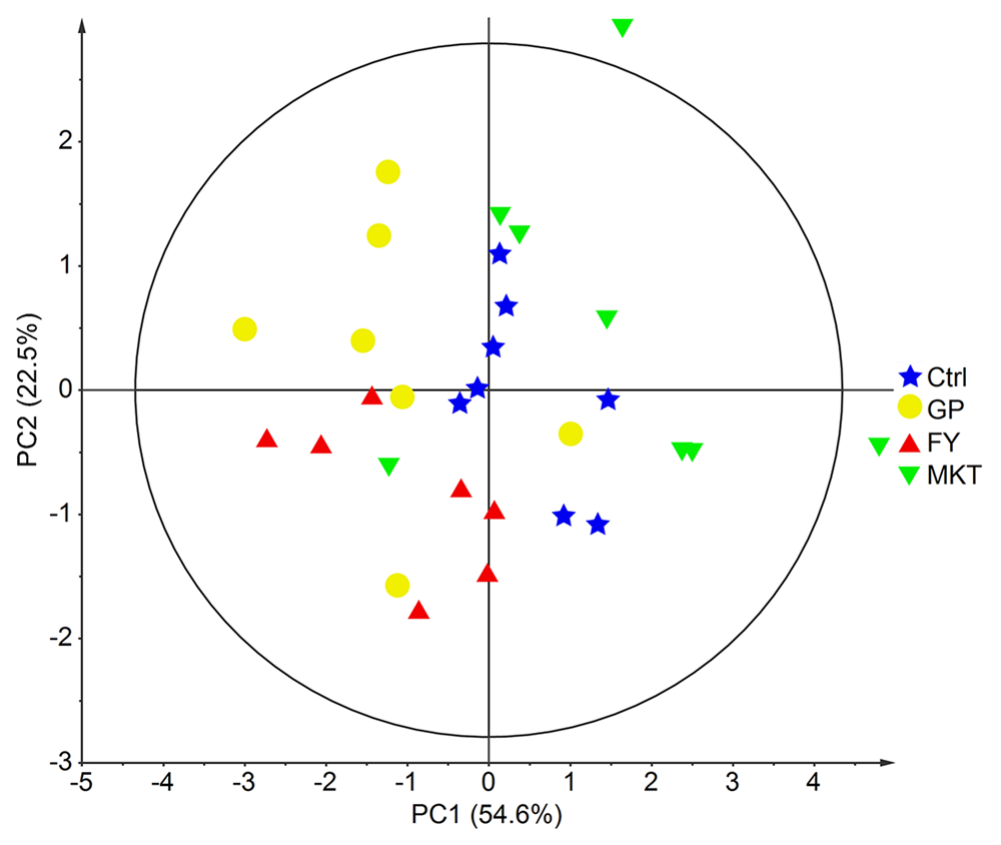
PCA based on 5 quantified muscle metabolites (cadaverine, carnitine, carnosine, NAD^+^ and nicotinurate). Data from dairy calves fed with control milk replacer (Ctrl, n = 8); milk replacer supplemented with glycine and proline (GP, n = 7); milk replacer supplemented with phenylalanine and tyrosine (FY, n = 7) and milk replacer supplemented with lysine, methionine and threonine (MKT, n = 8). Ellipse Hotelling’s T2 (95%).

Finally, when performing a PCA analysis based on the metabolites cadaverine, carnitine, carnosine, NAD^+^ and nicotinurate, Ctrl and MKT groups tend to appear on one side while GP and FY groups on the other side (Fig. 5).

## Discussion

The supplementation of MR with AAs is an important topic in pre-ruminant nutrition. In the present work, the impacts of supplementing MR with three different AA combinations (GP, FY and MKT) on weight gain, blood biochemistry profile and muscle metabolome were analysed after feeding the calves with control or supplemented MR for 7 weeks. The effectiveness of the supplementation has been evaluated by quantifying the plasma free AA profiles. Methionine/lysine/threonine, as well as phenylalanine, increased in the plasma of the supplemented groups as expected (MKT and FY, respectively). On the contrary, there were no significant differences in glycine/proline, and in tyrosine, probably due to the non-essential character of these AA or a difference in doses or intestinal absorption. An increase in plasma arginine has also been observed in the MKT group. A plausible explanation is that arginine and lysine share the same transport system for entering into the cell^23^. Thus, an increase in plasma lysine could compete with arginine for transport into the muscle leading to a concomitant increase in plasma arginine.

The profile of serum biochemical analytes considered as markers of the nutritional status of the animal (glucose, urea, creatinine, cholesterol, TGs, NEFAs and total protein) did not show major differences between groups. The only significant difference was observed in calves with MKT treatment, which showed higher serum values for creatinine versus the Ctrl group in the *t*-test and versus the FY group in the Tukey HSD test. These results suggest an increase in protein anabolism since blood creatinine concentration is positively related to muscular mass^24^. This conclusion may be reinforced by the observed tendency towards an increase in total protein in the MKT group versus the Ctrl group (*p* = 0.059). The enzymes ALT, AST and GGT were analysed for their role in the hepatic metabolism of AA and as markers of liver function, and no differences were observed between groups. Haptoglobin, marker of inflammation, and insulin and IGF-I, markers of metabolism and growth, were not altered either by AA supplementation.

The semitendinosus muscle NMR-metabolome was then characterized. From a glimpse of the results of muscle profile (Fig. 5), a remarkable finding is that the most distinguishable groups from Ctrl were FY and GP, in contrast with the ADG and serum analysis where MKT was the most divergent. Comparing Ctrl and MKT groups, there were no differences from the *t*-test and Tukey HSD pairwise comparisons of quantified muscle metabolites. Furthermore, the multivariate PLS-DAs of quantified metabolites and chemometrics results did not show an acceptable discrimination between Ctrl and MKT groups.

On the other hand, when comparing quantified metabolites in GP and FY groups versus Ctrl, the *t*-test identified nicotinurate being higher in FY and GP, carnosine being higher in GP and cadaverine being lower in FY. Differences between all groups are shown in Fig. 4, which summarizes the results from the ANOVA analysis with Tukey HSD test over the whole dataset. As explained in the figure, it is evident that GP and FY are different from MKT. Thus, cadaverine was lower in FY versus MKT, carnosine was higher in FY versus MKT. Nicotinurate was higher in GP versus Ctrl and MKT, and NAD^+^ was higher in GP and FY versus MKT. Furthermore, PLS-DAs of quantified metabolites and chemometrics shows an acceptable discrimination test between Ctrl-FY and FY-MKT comparison and PCA analysis based on the metabolites cadaverine, carnitine, carnosine, NAD^+^ and nicotinurate, grouped Ctrl and MKT group on one side and GP and FY groups on the other side (Fig. 5). Finally, when the surroundings of the two chemical shifts of cadaverine were analysed (between 1.422–1.467 ppm and 1.632–1.760 ppm), an increase in the total area in MKT group was observed versus both GP and FY groups. The increased total areas in MKT group may due to higher concentration of lysine and/or any of these other metabolites.

In summary, the main differential muscle metabolites were cadaverine, carnosine, carnitine, nicotinurate and NAD^+^. Considering the physiological interpretation of these results, cadaverine is the product of decarboxylation of lysine. Given the fact that MKT group was supplemented with lysine, this may be the reason for higher cadaverine levels.

Carnosine is a dipeptide abundant in skeletal muscle of mammals^25–27^. Amongst other biological functions, it is a powerful antioxidant^28,29^. It is interesting to notice that the conversion of proline into hydroxyproline during collagen synthesis requires the action of proline hydroxylase, an enzyme that needs molecular O_2_ as substrate. Likewise, the conversion of phenylalanine into tyrosine requires oxygen as a substrate for phenylalanine hydroxylase. This situation may indicate that the muscle cell in the groups supplemented with GP or FY could be under a situation of oxidative damage. The increase in carnosine concentration could alleviate this condition.

Carnitine is involved in the transport of acyl groups to the mitochondria, and thus in oxidative metabolism. This metabolite was also increased in GP. Similarly, nicotinurate, one of the metabolites of NAD^+^, is increased in the GP versus Ctrl and MKT groups. NAD^+^ is the final product of the oxidative respiratory chain and it has increased concentrations in both GP and FY groups.

Altogether, these results suggest a higher importance of aerobic metabolism in GP and FY groups.

Despite the fact that this study reveals the metabolomic changes due to the AA supplementation adaptation, two main limitations need however to be addressed. Firstly, this study is only focused on muscle metabolome, thus the general metabolomic profile is not presented. These results may be extended by analyzing the urine and/or serum metabolome. Secondly, only the aqueous fraction was analysed in the NMR. As such, organic phase remained unstudied. As a result, the lipid metabolism is not included and may omit some important information. Addressing these two points are important follow-up studies.

## Conclusions

To the best of our knowledge, this work is the first to report a NMR metabolomics study of skeletal muscle of calves with diets differing in AA supplementation. Herein we conclude that the AA supplementation added to the MR in the conditions given in the present work does not have a marked influence in the ADG in calves from 3 days to 7 weeks of age. Nevertheless, calves supplemented with MKT had higher serum creatinine concentration, considered an indicator of increased muscular mass. These differences between MKT and the Ctrl group are however not reflected in the metabolomics analysis of skeletal muscle, as all profiles are very similar. Nevertheless, the metabolomic analysis of skeletal muscle revealed several differences between the GP/FY groups and the Ctrl/MKT groups, suggesting a metabolic adaptation especially in the GP ad FY groups. In conclusion, our results support the performance improvement after MKT supplementation in MR compared with Ctrl and GP and FY combinations, and reveal that different AA profile of calf MR can modify protein and muscle metabolism. This study may help the dairy industry to take metabolic factors into account when supplementing relevant AAs in MR.

## Methods

### Animals and housing

Animals were managed according to the recommendations of the Animal Care Committee of *Institut de Recerca i Tecnologia Agroalimentàries* (IRTA) under the approval research protocol FUE-2017-00587321, authorization code 9733. Thirty-two Holstein male calves (weighing 44.6 ± 1.07 kg of BW at the age of 2.7 ± 0.27 days) were obtained from Granja Murucuc (Gurb, Barcelona, Spain) and housed individually at IRTA Torre Marimon (Caldes de Montbui, Barcelona, Spain). Animals used in the component here described were a part of a larger study with a higher number of animals that aimed to evaluate calf performance. The sample size was chosen based on the previous similar studies^18,19,21,30–32^.

### Diet treatments and performance recordings

MR was offered in 3-L nipple bottles twice a day, and all animals followed the same MR feeding program. Calves were fed on 4 L/d commercial milk replacer at 12.5% Dry Matter (DM). Milk replacer (Nukamel, Weert, The Netherlands) contained 25.4% CP (based on skimmed milk protein, and whey protein concentrate) and 20.3% fat from experimental days 1 to 4. Subsequently, the volume of MR was increased to 5 L/d from day 5 to 7, followed by 6 L/d from days 8 to 14, and 6 L/d at 15% DM concentration from day 15 to 49. At day 50, MR was reduced to one feeding per day of 3 L at 15% DM until day 56 when animals were fully weaned.

Calves were divided in four different treatments according to AA supplementation in the MR formula (which replaced whey protein concentrate in the control MR). All AAs were provided by Livzon Group (Fuxing Pharmaceutical, Ningxia (China)). Treatments were previously defined in a list, which indicated the MR treatment in each individual calf box. Then, as calves were arriving to the facilities, they were allocated correlatively to the individual boxes with a predetermined treatment. In this way, the only identification of the calves was the eartag number, which allowed the blinding of researchers and care-takers to treatment allocation. The four treatments were:

A (Ctrl): Control group with no AA supplementation

B (GP): supplementation with 0.1% glycine plus 0.3% proline in the MR formula

C (FY): supplementation with 0.2% phenylalanine plus 0.2% tyrosine in the MR formula

D (MKT): supplementation with 0.62% lysine plus 0.22% methionine plus 0.61% threonine in the MR formula

Ingredients and chemical composition are shown in Table 4. All animals had free access to water, and a pelleted concentrate starter feed (Pinallet, Cardona, Spain) was limited to minimize the impact of concentrate feeding on amino acid absorption: 100 g/day from day 1 to day 17 of study, 200 g/d from days 18 to 21, 300 g/d from day 22 to day 24. Subsequently, 400 g/d were used from day 25 to day 28, 500 g/d, from day 29 to day 31, 600 g/d from day 32 to day 33 d, 700 g/d at day 34, 800 g/d at day 35, 1,000 g/d from day 36 to day 42, 1,200 g/d from day 43 to day 49, and *ad libitum* afterwards until 63 d of study, when the study finished. Information about composition and AA concentration in the starter is provided in Table 5. Chopped barley straw was offered *ad libitum* during this study (Table 6). Calves were weighed weekly with an electronic scale (Mobba SC-01, Badalona, Spain) until the end of the study.

**Table 4:** Ingredients and chemical compositions of milk replacers.

Ingredients (g/kg DM)	Ctrl	GP	FY	MKT
Whey protein concentrate 35^a^	150	150	157	156
Skimmed milk powder	390	400	400	400
Fatted whey 50^b^	390	390	388.5	390
Whey protein concentrate 60^c^	40	26	20.5	9.5
Premix^d^	30	30	30	30
Proline	0	3	0	0
Glycine	0	1	0	0
Phenylalanine	0	0	2	0
Tyrosine	0	0	2	0
Lysine	0	0	0	6.2
Methionine	0	0	0	2.2
Threonine	0	0	0	6.1
**Composition, (g/kg DM, unless otherwise stated)**				
DM (g/kg)	979	974	981	980
CP	254	255	251	254
EE	202	204	202	202
Lactose	448	454	456	444
Ashes	70	73	70	69

Key: Ctrl: Control milk replacer; GP: Milk replacer supplemented with glycine and proline; FY: Milk replacer supplemented with phenylalanine and tyrosine and MKT: Milk replacer supplemented with lysine, methionine and threonine. CP = crude protein; DM = dry matter; EE = ether extract.

^a^Contains 35% protein in the whey.

^b^Contains 50% protein in the whey.

^c^Contains 60% protein in the whey.

^d^Mineral and vitamin composition: Vitamin A 25,000 IU/kg; Vitamin D3 4,500 IU/kg; Vitamin E 300 mg/kg, Vitamin C 300 mg/kg, Vitamin B1 16 mg/kg, Vitamin B2 10 mg/kg, Vitamin B6 10 mg/kg, Vitamin B12 80 mg/T, Vitamin K 5.5 mg/kg, Folic acid 1 mg/kg, Pantothenic acid 23 mg/kg, Niacin 50 mg/kg, Fe 150 mg/kg, Zinc 170 mg/kg, Copper 10 mg/kg, Manganese 40 mg/kg, Iodine 1.3 mg/kg, Selenium 0.4 mg/kg.

**Table 5:** Chemical omposition and aminoacid content in the starter feed.

Composition	
DM (%)	87.55
CP (% DM)	17.85
**Detailed Amino Acid Composition (g/kg DM)**	
Aspartic acid	1.55
Glutamic acid	3.33
Serine	0.85
Histidine	0.41
Glycine	0.76
Threonine	0.7
Arginine	1.17
Alanine	0.79
Tyrosine	0.58
Valine	0.77
Methionine	0.21
Phenylalanine	0.82
Isoleucine	0.66
Leucine	1.35
Lysine	1.06
Hydroxyproline	0.043
Proline	1.05
Tryptophan	0.19

CP = crude protein; DM = dry matter.

**Table 6:** Chemical composition of the barley chopped straw.

Composition	
DM (%)	83.8
CP (% DM)	3.8
ADF (% DM)	52.9
NDF (% DM)	79.7
Ash content (% DM)	6.4

CP = crude protein; DM = dry matter; ADF = Acid Detergent Fibre; NDF = Neutral Detergent Fibre.

### Blood and muscle sample collection

At the 7th week of the study, blood samples were obtained from jugular vein 2 h after the morning MR feeding, and were kept in 10 ml Vacutainer tubes without anticoagulant to obtain the serum for biochemistry profiles or with lithium heparin for plasma AA analyses. Serum and plasma were obtained by centrifugation at 1,500 *g* for 10 min and stored in aliquots at −20 °C until further analysis.

At 47.9 ± 0.46 days of age, a biopsy was obtained from semitendinosus muscle. Briefly, animals were injected with xylazine intravenously at the dose of 0.005 mL/kg BW, once the animals were sedated, the hind leg was shaved and disinfected with iodine povidone. Then, 2 mL of 0.02 g/mL lidocaine were subcutaneously injected as a local anaesthetic. A 3-cm cut in the skin and subcutaneous layer was done with a scalpel, and a 2-cm zone of the muscle was delimited with suture, and it was dissected with curve scissors. Finally, the muscular, subcutaneous and cutaneous layers were sutured. Muscle samples were immediately frozen into liquid nitrogen and subsequently kept at −80 °C until further analysis.

### Quantification of plasma AAs

Plasma AAs were measured by HPLC following a modified protocol^33^. At the day of assay, 100 µl of thawed plasma were transferred to a 2.0 mL micro-centrifuge tube and 10 µl of 10 mM DTT in PBS (pH 7.4) and 5,79 µl 2.5 mM GABA as internal standard were added. The solution was vortexed for 30 s and an equivalent volume of 10% sulfosalicylic acid was added to precipitate the proteins, followed by mixing for 2 min. The samples were centrifuged at 14,000 g for 10 min at 4 °C and the supernatant collected^34^.

For derivatization, the AccQ Fluor (Waters, Milford, MA, USA) method was used following the instructions provided by the manufacturer. A total of 10 μL standard solution or supernatant of deproteinized plasma sample was taken into a 1.5 mL micro-centrifuge tube, buffered with 70 µL Waters AccQFluor Borate Buffer and derivatized by addition of 20 µL of AccQ-Fluor reagent, vortexed and heated for 10 min at 55 °C.

HPLC was performed on an Elite LaCHrom (Hitachi, Tokyo, Japan) equipped with an UV detector (Hitachi L-24200, Tokyo, Japan) with a Novapak C18 column (300 mm × 3.9 mm) from Waters. The flow rate was 1.0 ml/min and the column temperature was kept at 28 °C. The injection volume was 10 μL and the detection wavelength was set at 254 nm. The solvent system consisted of two eluents: (A) AccQ > Tag eluent A concentrate (10%, v/v) and water (90%, v/v) and (B) 60% acetonitrile/40% water. The following gradient elution was used: 0 to 0.50 min, 100% A; 0.5 min, 98% A to 2% B; 20 min, 93% A to 7% B; 32 min, 91.5% A to 8.5% B; 40 min, 82% A to 18% B; 47.5 min: 79% A to 21% B; 55 min: 60% A to 40% B; 60 min: 100% B. Pure AAs were used as standards and GABA was used as internal control (Sigma, St. Louis, MO, USA). Standards were prepared at 2.5 mM stock solutions in 0.1 M HCl and was serially diluted from 500 µM to 7.81 µM to perform the standard curve in order to quantify AA of plasma samples. EZChrom Elite system V3.1.7 software (Agilent, Santa Clara, CA, USA) was used for system control and data acquisition. Validation of the technique showed a good precision (Coefficients of Variation from 1% to 5% for all AA), good linearity (R^2^ > 0.998) except for serine (R^2^ = 0.990) and cysteine (R^2^ = 0.989) and a limit of quantification lower than 10 μM (except for serine (11 μM) and histidine/glutamine (21 μM).

### Determination of serum biochemical analytes

Serum biochemistry analytes were analysed with the Olympus AU400 analyser with following techniques and OSR reagents (Olympus System Reagent)^35,36^: glucose (HK method), urea (GLDH method), creatinine (Jaffé method), cholesterol (CHOP-PAP-method), triglyceride (GPO-PAP method), total protein (Biuret method), ALT (International Federation of Clinical Chemistry (IFCC) method), AST (IFCC method) and GGT (IFCC method). NEFAs were determined with NEFA-C reagent (Wako Chemicals GmbH, Neuss, Germany). Quality control protocols were based on the daily quantification of two control sera of low and high concentration (Control serum I and Control serum II, Beckman Coulter) Haptoglobin was determined by a colorimetric method (Tridelta, Ireland). Insulin and IGF-I were determined by ELISA (Bovine Insulin ELISA from Mercodia, Uppsala, Sweden and Human IGF-I ELISA E20 from Mediagnost, Reutlingen, Germany). This reagent is suitable for bovine samples, as declared by the manufacturer, but the cross-reactivity was not assessed by the authors.

Intra-assay Coefficients of Variation (CV) were: glucose (1.7%), urea (2.1%), creatinine (1.4%), cholesterol (1.1%), triglycerides (3.0%), total protein (0.9), ALT (1.7%), AST (1.0%), GGT (0.6%), NEFAs (2.7%), Haptoglobin (4.1%), insulin (4.1%) and IGF-1 (4.4%).

### Muscle extraction and NMR data acquisition

Muscle tissues were transferred from −80 °C freezer to dry ice. All muscle tissues were ground into fine powder while mixed with liquid nitrogen in a mortar and pestle. The extraction of the aqueous metabolites was performed using a modified chloroform/methanol method previously described^21^. In brief: approximately 0.5 g of muscle tissue was mixed with 1.2 mL of chloroform/methanol (1:2 v/v) in a glass tube and was vortexed for 1 min. Then an extra 0.4 mL of chloroform was added to compensate the evaporated chloroform and the tube was vortexed for an additional 1 minute. Afterwards 0.4 mL of double distilled water was added and the tube vortexed again for 1 min. The homogenate was centrifuged at 1935 *g* for 20 min at 4 °C. 1000 μL of the top (methanol/water) fraction was transferred into a 2 mL polypropylene tube and dried in a centrifugal vacuum concentrator. To resuspend the dried sample for NMR analysis, 600 µL of phosphate buffer in D_2_O (150 mM; pH 6.6 (pD 7.0); with 100 μM of TSP-D4 (deuterated Trimethylsilylpropanoic acid)) were added, followed by 1 min vortexing and 10 min centrifugation at 14,100 g at room temperature. The supernatants were transferred into NMR borosilicate tubes rated for 800 MHz (New Era, Vineland, NJ, USA).

1D ^1^H NOESY spectra were collected on an 800 MHz NMR spectrometer equipped with a room temperature HCN inverse Z-gradient probe (Bruker Biospin, Billerica, MA, USA). The pulse sequence used for 1D gradient NOESY with water pre-saturation was “noesygppr1D” with the Bruker standard acquisition parameters for profiling (spectral width: 16393.443 Hz, recycling delay: 4 s, acquisition time: 1.9988480 s, mixing time 10 ms). 96 scans and 8 dummy scans were used for each spectrum resulting in a 10-min experiment. For select samples, additional homonuclear and heteronuclear spectra (^1^H J-resolved, ^1^H-^1^H COSY, and ^1^H-^13^C HSQC) spectra were also collected to assist with compound identification.

### NMR data processing and analysis

Spectra were processed with 1 Hz exponential appodization, no additional zero filling and polynomial baseline correction with the TopSpin 3.2 software (Bruker Biospin, Billerica, MA, USA). Compound quantification in reference to the TSP internal standard was performed with the Chenomx 8.1 NMR Suit (Edmonton, Alberta, Canada). The limit used for compound quantification in Chenomx was set at 0.002 mM.

Metabolomics data have been deposited to the EMBL-EBI MetaboLights database (DOI: 10.1093/nar/gks1004. PubMed PMID: 23109552) with the identifier MTBLS720. The complete dataset can be accessed here https://www.ebi.ac.uk/metabolights/MTBLS720.

### Statistical analysis

#### Performance and blood data

The univariate analysis was carried out with the SPSS 24 (IBM, Armonk, NY, USA). The significance level was declared at *P* < 0.05 and a tendency was considered at 0.05 ≤ *P* ≤ 0.1. Descriptive data are presented with the means and the standard error (mean ± SE). Shapiro-Wilks test was used to assess normality of data. Means were compared to the Ctrl group by a *t*-test and for pairwise comparisons, ANOVA with Tukey HSD post hoc was applied. The multivariate analyses were performed by SIMCA 13.0.3 (Umetrics AB, Umeå, Sweden) after applying auto-transform to reduce data skewness and UV scaling to reduce relative importance of large values.

#### Muscle data

For Chemometric statistical analysis, processed spectra were loaded into “R” where peak alignment was performed with SPEAQ. The data were then exported into a coma-separated value (CSV) file and loaded into SIMCA 13.0.3. All spectra were treated with a log transform and by Pareto scaling. For the whole groups, unsupervised Principal Components Analysis (PCA) was performed to detect outliers, patterns and trends. For pairwise Partial Least Square Discriminant Analyses (PLS-DAs), Q^2^ > 0.4 were selected as acceptable models and Variable Importance in Projection (VIP) scores were used to identify metabolites responsible for the separation. These metabolites together with others that are relevant for the project were then quantified using database assisted spectral deconvolution that was performed using Chenomx. The resulting concentration were normalized based on the mass tissue used for extraction and then further performed univariate analysis by SPSS 24 and multivariate analysis by SIMCA 13.0.3 after applying auto-transform and UV scaling.

## Electronic supplementary material

Supplementary material

## Data Availability

All data generated or analysed during this study are included in this published article and its Supplementary files.
