# Supplementary material for: Skeletal muscle metabolomics and blood biochemistry analysis reveal metabolic changes associated with dietary amino acid supplementation in dairy calves
Source: Sci Rep. 2018 Sep 14;8:13850. doi: 10.1038/s41598-018-32241-4 (PMC6138728; doi:10.1038/s41598-018-32241-4)
Supplement: Supplementary file 1 — Supplementary material [file 41598_2018_32241_MOESM1_ESM.pdf]

Supplementary Material

Skeletal muscle metabolomics and blood biochemistry analysis reveal metabolic changes associated with dietary amino acid supplementation in dairy calves

Kuai Yu [1], Manolis Matzapetakis [3], Daniel Valent [2], Yolanda Saco [1,2], Andre M. Almeida [4], Marta Terré [5] and Anna Bassols [1,2]

Supplementary Figure S1: a: Binned muscle extract NMR spectra between 1.422 - 1.467 and 1.632 - 1.760 ppm; **b** and **c:** Total area quantification between 1.422 - 1.467 and 1.632 -1.760 ppm, respectively. Page 2

Supplementary Table S 1: List of 70 identified metabolites in muscle tissue. Page 3

Supplementary Table S2: VIP score>2.5 with metabolite assignments of chemometrics PLS-DA in Ctrl - FY comparison. Page 4

Supplementary Table S3 Tukey HSD of ANOVA for quantified blood analytes from calves supplemented with different amino acids. Page 10

Supplementary Table S4 Tukey HSD of ANOVA for quantified muscle metabolites from calves supplemented with different amino acids. Page 18

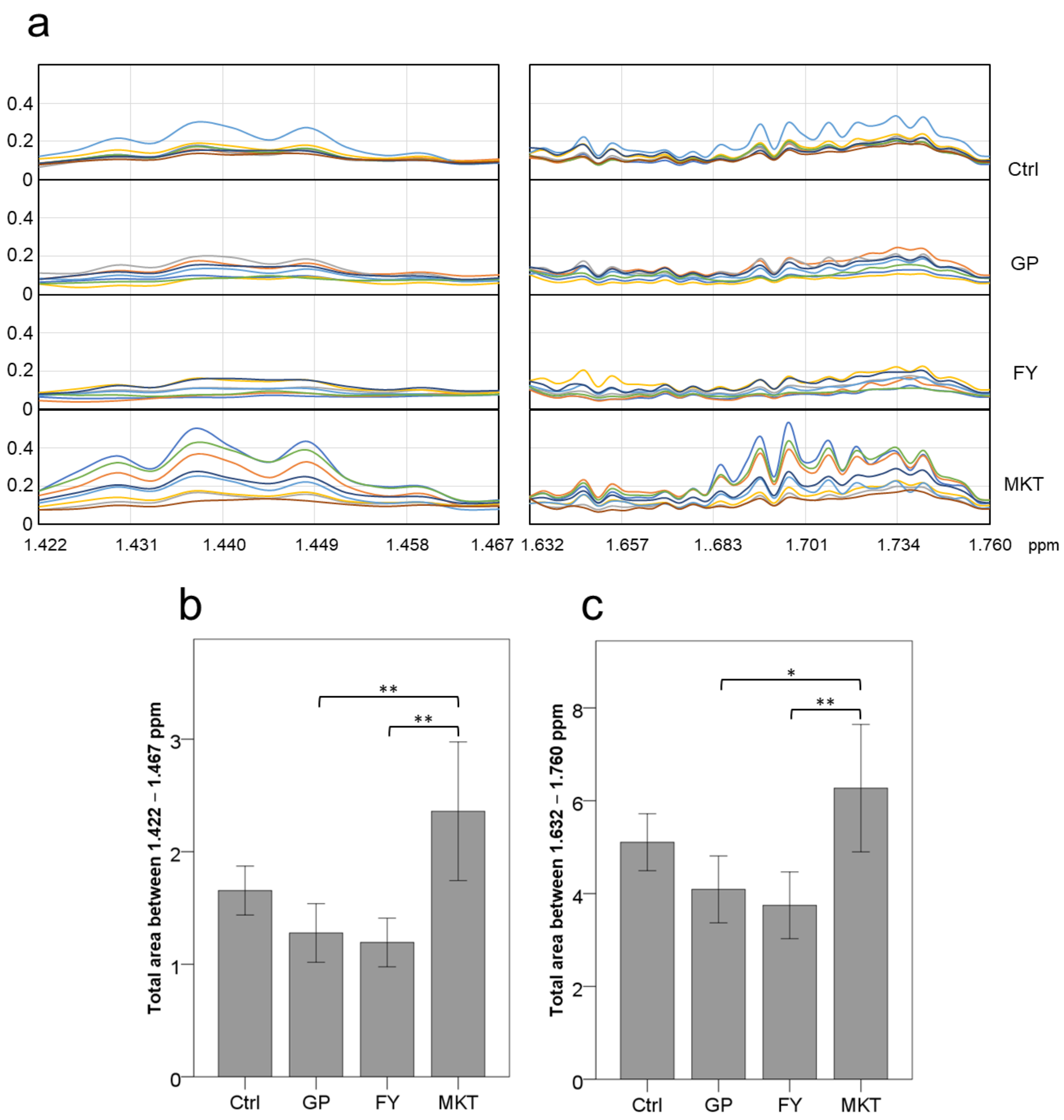
a
0.4
0.2
0
Ctrl
GP
FY
MKT
1.422
1.431
1.440
1.449
1.458
1.467
1.632
1.657
1..683
1.701
1.734
1.760
ppm
b
Total area between 1.422 – 1.467 ppm
**
**
Ctrl
GP
FY
MKT
c
Total area between 1.632 – 1.760 ppm
*
**
Ctrl
GP
FY
MKT

Supplementary Table S1: List 70 Identified muscle tissue metabolites. Key: Quantified metabolites are shown in bold.

**3-Hydroxybutyrate**	**Acetate**	Acetoacetate	Adenine
**ADP**	**Alanine**	**AMP**	**Anserine**
Arginine	Asparagine	**Aspartate**	**ATP**
**Betaine**	**Cadaverine**	**Carnitine**	**Carnosine**
Citrate	**Creatine**	**Creatine phosphate**	**Creatinine**
Cysteine	Cystine	D-Lactic acid	Ethanol
Formate	**Fumarate**	**Glucose**	Glucose-1-phosphate
**Glucose-6-phosphate**	**Glutamate**	Glutamine	Glutathione
Glycerol 3-phosphate	**Glycine**	GTP	Histamine
Histidine	Homocysteine	IMP	Inosine
**Isoleucine**	**Lactate**	**Leucine**	Lysine
Malonic acid	**Mannose**	Methanol	Methionine
**Myo-Inositol**	**NAD+**	**NADH**	**NADP**
NADPH	**Nicotinurate**	**O-Acetylcarnitine**	O-Phosphocholine
Ornithine	**Phenylalanine**	Proline	**Pyruvate**
Serine	**Taurine**	Threonine	**Tryptophan**
**Tyrosine**	UMP	Uridine	**Valine**
π-Methylhistidine	τ-Methylhistidine		

Table S2 Variable importance in projection (VIP) score>2.5 with metabolite assignments of chemometrics PLS discriminant analysis in Ctrl - FY comparison.

Chemical shi	VIP	Compound
1.418	2.66	Cadaverine/Lysine
1.418	2.69	Cadaverine/Lysine
1.418	2.71	Cadaverine/Lysine
1.419	2.72	Cadaverine/Lysine
1.419	2.72	Cadaverine/Lysine
1.419	2.70	Cadaverine/Lysine
1.419	2.69	Cadaverine/Lysine
1.420	2.68	Cadaverine/Lysine
1.420	2.67	Cadaverine/Lysine
1.420	2.67	Cadaverine/Lysine
1.421	2.66	Cadaverine/Lysine
1.421	2.64	Cadaverine/Lysine
1.421	2.61	Cadaverine/Lysine
1.422	2.60	Cadaverine/Lysine
1.422	2.59	Cadaverine/Lysine
1.422	2.58	Cadaverine/Lysine
1.423	2.57	Cadaverine/Lysine
1.423	2.55	Cadaverine/Lysine
1.423	2.51	Cadaverine/Lysine
1.429	2.52	Cadaverine/Lysine
1.430	2.57	Cadaverine/Lysine
1.430	2.59	Cadaverine/Lysine
1.430	2.59	Cadaverine/Lysine
1.431	2.58	Cadaverine/Lysine
1.431	2.55	Cadaverine/Lysine
1.431	2.50	Cadaverine/Lysine
1.664	2.70	Leucine/Unassigned 7
1.664	2.77	Leucine/Unassigned 7
1.664	2.83	Leucine/Unassigned 7
1.665	2.87	Leucine/Unassigned 7
1.665	2.88	Leucine/Unassigned 7
1.665	2.87	Leucine/Unassigned 7
1.666	2.85	Leucine/Unassigned 7
1.666	2.81	Leucine/Unassigned 7
1.666	2.77	Leucine/Unassigned 7
1.667	2.71	Leucine/Unassigned 7
1.667	2.71	Leucine/Unassigned 7
1.667	2.60	Leucine/Unassigned 7
1.668	2.52	Leucine/Unassigned 7
1.670	2.60	Leucine/Unassigned 7
1.671	2.73	Leucine/Unassigned 7
1.671	2.52	Leucine/Unassigned 7
1.672	2.59	Leucine/Unassigned 7
1.672	2.66	Leucine/Unassigned 7
1.672	2.72	Leucine/Unassigned 7

1.673	2.78	Leucine/Unassigned 7
1.673	2.63	Leucine/Unassigned 7
1.673	2.66	Leucine/Unassigned 7
1.674	2.69	Leucine/Unassigned 7
1.674	2.71	Leucine/Unassigned 7
1.674	2.72	Leucine/Unassigned 7
1.674	2.71	Leucine/Unassigned 7
1.675	2.70	Leucine/Unassigned 7
1.675	2.72	Leucine/Unassigned 7
1.675	2.78	Leucine/Unassigned 7
1.676	2.87	Leucine/Unassigned 7
1.676	2.95	Leucine/Unassigned 7
1.676	3.01	Leucine/Unassigned 7
1.677	3.01	Leucine/Unassigned 7
1.677	2.95	Leucine/Unassigned 7
1.677	2.89	Leucine/Unassigned 7
1.678	2.77	Leucine/Unassigned 7
1.678	2.68	Leucine/Unassigned 7
1.678	2.60	Leucine/Unassigned 7
1.679	2.57	Leucine/Unassigned 7
1.679	2.57	Leucine/Unassigned 7
1.679	2.61	Leucine/Unassigned 7
1.679	2.67	Leucine/Unassigned 7
1.680	2.75	Leucine/Unassigned 7
1.680	2.83	Leucine/Unassigned 7
1.680	2.90	Leucine/Unassigned 7
1.681	2.92	Leucine/Unassigned 7
1.681	2.91	Leucine/Unassigned 7
1.681	2.87	Leucine/Unassigned 7
1.682	2.82	Leucine/Unassigned 7
1.682	2.78	Leucine/Unassigned 7
1.682	2.74	Leucine/Unassigned 7
1.683	2.72	Leucine/Unassigned 7
1.683	2.72	Leucine/Unassigned 7
1.683	2.93	Leucine/Unassigned 7
1.684	2.99	Leucine/Unassigned 7
1.684	3.06	Leucine/Unassigned 7
1.684	3.11	Leucine/Unassigned 7
1.684	3.14	Leucine/Unassigned 7
1.685	3.15	Leucine/Unassigned 7
1.685	3.12	Leucine/Unassigned 7
1.685	3.06	Leucine/Unassigned 7
1.686	2.98	Leucine/Unassigned 7
1.686	2.89	Leucine/Unassigned 7
1.686	2.80	Leucine/Unassigned 7
1.687	2.70	Leucine/Unassigned 7
1.687	2.59	Leucine/Unassigned 7
1.690	2.50	Cadaverine/Lysine/Leucine
1.690	2.54	Cadaverine/Lysine/Leucine

1.691	2.61	Cadaverine/Lysine/Leucine
1.691	2.67	Cadaverine/Lysine/Leucine
1.691	2.71	Cadaverine/Lysine/Leucine
1.692	2.73	Cadaverine/Lysine/Leucine
1.692	2.72	Cadaverine/Lysine/Leucine
1.692	2.68	Cadaverine/Lysine/Leucine
1.693	2.63	Cadaverine/Lysine/Leucine
1.693	2.57	Cadaverine/Lysine/Leucine
1.693	2.51	Cadaverine/Lysine/Leucine
1.705	2.54	Cadaverine/Lysine/Arginine/Leucine
1.705	2.58	Cadaverine/Lysine/Arginine/Leucine
1.705	2.59	Cadaverine/Lysine/Arginine/Leucine
1.706	2.60	Cadaverine/Lysine/Arginine/Leucine
1.706	2.59	Cadaverine/Lysine/Arginine/Leucine
1.706	2.59	Cadaverine/Lysine/Arginine/Leucine
1.707	2.58	Cadaverine/Lysine/Arginine/Leucine
1.707	2.55	Cadaverine/Lysine/Arginine/Leucine
1.715	2.52	Cadaverine/Lysine/Arginine/Leucine
1.716	2.54	Cadaverine/Lysine/Arginine/Leucine
1.716	2.53	Cadaverine/Lysine/Arginine/Leucine
3.764	2.52	Lysine/Alanine/Mannose/Glutamine/Arginine/Glutathione/Ornithine/Glucose/Glucose-1-phosphate/π-Methylhistidine
3.764	2.83	Lysine/Alanine/Mannose/Glutamine/Arginine/Glutathione/Ornithine/Glucose/Glucose-1-phosphate/π-Methylhistidine
3.765	3.02	Lysine/Alanine/Mannose/Glutamine/Arginine/Glutathione/Ornithine/Glucose/Glucose-1-phosphate/π-Methylhistidine
3.765	3.02	Lysine/Alanine/Mannose/Glutamine/Arginine/Glutathione/Ornithine/Glucose/Glucose-1-phosphate/π-Methylhistidine
3.765	2.53	Lysine/Alanine/Mannose/Glutamine/Arginine/Glutathione/Ornithine/Glucose/Glucose-1-phosphate/π-Methylhistidine
4.683	2.56	NADH
4.683	2.59	NADH
4.684	2.52	NADH
5.451	2.51	Glucose-1-phosphate
5.452	2.74	Glucose-1-phosphate
5.452	2.82	Glucose-1-phosphate
5.452	2.77	Glucose-1-phosphate
5.453	2.59	Glucose-1-phosphate
5.456	2.60	Glucose-1-phosphate
5.457	2.67	Glucose-1-phosphate
5.457	2.60	Glucose-1-phosphate
5.715	2.57	Unassigned 4
5.715	2.59	Unassigned 4
5.912	2.54	Uridine
5.913	2.67	Uridine
5.913	2.71	Uridine
5.913	2.80	Uridine
5.914	2.91	Uridine

5.914	2.76	Uridine
5.914	2.55	Uridine
5.916	2.50	Uridine
5.917	2.63	Uridine
5.917	2.84	Uridine
5.917	2.88	Uridine
5.917	2.79	Uridine
5.918	2.75	Uridine
5.918	2.70	Uridine
5.918	2.60	Uridine
5.936	2.58	GTP
5.936	2.69	GTP
5.936	2.76	GTP
5.937	2.80	GTP
5.937	2.77	GTP
5.937	2.72	GTP
5.937	2.66	GTP
5.938	2.59	GTP
5.938	2.53	GTP
5.942	2.57	GTP
5.942	2.76	GTP
5.942	2.91	GTP
5.943	3.05	GTP
5.943	3.15	GTP
5.943	3.16	GTP
5.944	3.11	GTP
5.944	3.06	GTP
5.944	2.98	GTP
5.945	2.84	GTP
5.945	2.71	GTP
5.945	2.64	GTP
5.946	2.62	GTP
5.946	2.58	GTP
5.946	2.51	GTP
5.967	2.56	NADH/NADPH/UMP
5.967	2.57	NADH/NADPH/UMP
5.967	2.51	NADH/NADPH/UMP
5.971	2.54	NADH/NADPH/UMP
5.971	2.73	NADH/NADPH/UMP
5.972	2.88	NADH/NADPH/UMP
5.972	2.96	NADH/NADPH/UMP
5.972	2.99	NADH/NADPH/UMP
5.972	2.94	NADH/NADPH/UMP
5.973	2.83	NADH/NADPH/UMP
5.973	2.72	NADH/NADPH/UMP
5.973	2.65	NADH/NADPH/UMP
5.974	2.61	NADH/NADPH/UMP
5.974	2.59	NADH/NADPH/UMP
5.974	2.58	NADH/NADPH/UMP

5.975	2.59	NADH/NADPH/UMP
5.975	2.62	NADH/NADPH/UMP
5.975	2.66	NADH/NADPH/UMP
5.976	2.72	NADH/NADPH/UMP
5.976	2.79	NADH/NADPH/UMP
5.976	2.83	NADH/NADPH/UMP
5.977	2.84	NADH/NADPH/UMP
5.977	2.82	NADH/NADPH/UMP
5.977	2.75	NADH/NADPH/UMP
5.977	2.63	NADH/NADPH/UMP
5.978	2.53	NADH/NADPH/UMP
6.448	2.53	Unassigned 5
6.448	2.59	Unassigned 5
6.526	2.51	Fumarate
6.526	2.54	Fumarate
6.677	2.67	Unassigned 2
6.677	2.72	Unassigned 2
6.677	2.58	Unassigned 2
6.955	2.54	NADH
7.260	2.50	Tryptophan
7.260	2.56	Tryptophan
7.260	2.59	Tryptophan
7.261	2.59	Tryptophan
7.261	2.57	Tryptophan
7.261	2.52	Tryptophan
8.013	2.51	Unassigned 6
8.013	2.54	Unassigned 6
8.586	2.65	Unassigned 1
8.586	2.76	Unassigned 1
8.587	2.75	Unassigned 1
8.587	2.76	Unassigned 1
8.587	2.83	Unassigned 1
8.588	2.91	Unassigned 1
8.588	2.98	Unassigned 1
8.588	2.97	Unassigned 1
8.589	2.87	Unassigned 1
8.589	2.74	Unassigned 1
8.589	2.65	Unassigned 1
8.589	2.63	Unassigned 1
8.590	2.63	Unassigned 1
8.590	2.60	Unassigned 1
8.590	2.50	Unassigned 1
8.922	2.57	Nicotinurate
8.922	2.64	Nicotinurate
8.922	2.62	Nicotinurate
8.923	2.50	Nicotinurate
8.924	2.71	Nicotinurate
8.924	2.81	Nicotinurate
8.924	2.79	Nicotinurate

8.925	2.86	Nicotinurate
8.925	2.96	Nicotinurate
8.925	3.02	Nicotinurate
8.925	3.03	Nicotinurate
8.926	3.01	Nicotinurate
8.926	2.93	Nicotinurate
8.926	2.81	Nicotinurate
8.927	2.81	Nicotinurate
8.927	2.89	Nicotinurate
8.927	2.87	Nicotinurate
8.928	2.84	Nicotinurate
8.928	2.87	Nicotinurate
8.928	2.94	Nicotinurate
8.929	3.05	Nicotinurate
8.929	3.08	Nicotinurate
8.929	3.00	Nicotinurate
8.930	2.89	Nicotinurate
8.930	2.77	Nicotinurate
8.930	2.68	Nicotinurate
8.930	2.64	Nicotinurate
8.931	2.60	Nicotinurate
8.931	2.57	Nicotinurate
8.931	2.53	Nicotinurate
9.002	2.50	Unassigned 3
9.002	2.67	Unassigned 3
9.002	2.58	Unassigned 3

Table S3 Tukey HSD of ANOVA for quantified blood parameters from calves supplemented with different amino acids.

Dependent Variable			Mean Difference (I-J)	Std. Error	Sig.	95% Confidence Lower Bound	Upper Bound
Glucose	Ctrl	FY	-10.638	6.841	0.420	-29.316	8.041
		GP	-5.975	6.841	0.818	-24.653	12.703
		MKT	-6.525	6.841	0.776	-25.203	12.153
	FY	Ctrl	10.638	6.841	0.420	-8.041	29.316
		GP	4.663	6.841	0.903	-14.016	23.341
		MKT	4.113	6.841	0.931	-14.566	22.791
	GP	Ctrl	5.975	6.841	0.818	-12.703	24.653
		FY	-4.663	6.841	0.903	-23.341	14.016
		MKT	-0.550	6.841	1.000	-19.228	18.128
	MKT	Ctrl	6.525	6.841	0.776	-12.153	25.203
		FY	-4.113	6.841	0.931	-22.791	14.566
		GP	0.550	6.841	1.000	-18.128	19.228
Urea	Ctrl	FY	0.700	1.509	0.966	-3.421	4.821
		GP	-2.863	1.509	0.252	-6.983	1.258
		MKT	-1.088	1.509	0.888	-5.208	3.033
	FY	Ctrl	-0.700	1.509	0.966	-4.821	3.421
		GP	-3.563	1.509	0.109	-7.683	0.558
		MKT	-1.788	1.509	0.641	-5.908	2.333
	GP	Ctrl	2.863	1.509	0.252	-1.258	6.983
		FY	3.563	1.509	0.109	-0.558	7.683
		MKT	1.775	1.509	0.647	-2.346	5.896
	MKT	Ctrl	1.088	1.509	0.888	-3.033	5.208
		FY	1.788	1.509	0.641	-2.333	5.908
		GP	-1.775	1.509	0.647	-5.896	2.346
Creatinine	Ctrl	FY	0.034	0.052	0.913	-0.107	0.175
		GP	-0.078	0.052	0.450	-0.218	0.063
		MKT	-0.109	0.052	0.175	-0.250	0.032
	FY	Ctrl	-0.034	0.052	0.913	-0.175	0.107
		GP	-0.111	0.052	0.160	-0.252	0.030
		MKT	-.14250*	0.052	0.047	-0.283	-0.002
	GP	Ctrl	0.078	0.052	0.450	-0.063	0.218
		FY	0.111	0.052	0.160	-0.030	0.252
		MKT	-0.031	0.052	0.929	-0.172	0.110
	MKT	Ctrl	0.109	0.052	0.175	-0.032	0.250
		FY	.14250*	0.052	0.047	0.002	0.283
		GP	0.031	0.052	0.929	-0.110	0.172
Cholesterol	Ctrl	FY	-3.038	12.550	0.995	-37.303	31.228
		GP	-0.263	12.550	1.000	-34.528	34.003
		MKT	-7.725	12.550	0.926	-41.990	26.540
	FY	Ctrl	3.038	12.550	0.995	-31.228	37.303
		GP	2.775	12.550	0.996	-31.490	37.040

		MKT	-4.687	12.550	0.982	-38.953	29.578
	GP	Ctrl	0.263	12.550	1.000	-34.003	34.528
		FY	-2.775	12.550	0.996	-37.040	31.490
		MKT	-7.462	12.550	0.933	-41.728	26.803
	MKT	Ctrl	7.725	12.550	0.926	-26.540	41.990
		FY	4.687	12.550	0.982	-29.578	38.953
		GP	7.462	12.550	0.933	-26.803	41.728
TGs	Ctrl	FY	3.713	6.357	0.936	-13.644	21.069
		GP	-1.075	6.357	0.998	-18.432	16.282
		MKT	-3.600	6.357	0.941	-20.957	13.757
	FY	Ctrl	-3.713	6.357	0.936	-21.069	13.644
		GP	-4.788	6.357	0.875	-22.144	12.569
		MKT	-7.313	6.357	0.662	-24.669	10.044
	GP	Ctrl	1.075	6.357	0.998	-16.282	18.432
		FY	4.788	6.357	0.875	-12.569	22.144
		MKT	-2.525	6.357	0.978	-19.882	14.832
	MKT	Ctrl	3.600	6.357	0.941	-13.757	20.957
		FY	7.313	6.357	0.662	-10.044	24.669
		GP	2.525	6.357	0.978	-14.832	19.882
NEFAs	Ctrl	FY	0.014	0.036	0.980	-0.084	0.111
		GP	-0.013	0.036	0.985	-0.110	0.085
		MKT	-0.005	0.036	0.999	-0.103	0.093
	FY	Ctrl	-0.014	0.036	0.980	-0.111	0.084
		GP	-0.026	0.036	0.883	-0.124	0.071
		MKT	-0.019	0.036	0.953	-0.116	0.079
	GP	Ctrl	0.013	0.036	0.985	-0.085	0.110
		FY	0.026	0.036	0.883	-0.071	0.124
		MKT	0.008	0.036	0.997	-0.090	0.105
	MKT	Ctrl	0.005	0.036	0.999	-0.093	0.103
		FY	0.019	0.036	0.953	-0.079	0.116
		GP	-0.008	0.036	0.997	-0.105	0.090
Total Protein	Ctrl	FY	-0.173	0.183	0.782	-0.672	0.327
		GP	-0.026	0.183	0.999	-0.526	0.473
		MKT	-0.353	0.183	0.240	-0.852	0.147
	FY	Ctrl	0.173	0.183	0.782	-0.327	0.672
		GP	0.146	0.183	0.854	-0.353	0.646
		MKT	-0.180	0.183	0.760	-0.680	0.320
	GP	Ctrl	0.026	0.183	0.999	-0.473	0.526
		FY	-0.146	0.183	0.854	-0.646	0.353
		MKT	-0.326	0.183	0.302	-0.826	0.173
	MKT	Ctrl	0.353	0.183	0.240	-0.147	0.852
		FY	0.180	0.183	0.760	-0.320	0.680
		GP	0.326	0.183	0.302	-0.173	0.826
ALT	Ctrl	FY	1.163	1.490	0.863	-2.905	5.230
		GP	-0.675	1.490	0.968	-4.742	3.392
		MKT	0.238	1.490	0.999	-3.830	4.305
	FY	Ctrl	-1.163	1.490	0.863	-5.230	2.905

		GP	-1.838	1.490	0.611	-5.905	2.230
		MKT	-0.925	1.490	0.924	-4.992	3.142
	GP	Ctrl	0.675	1.490	0.968	-3.392	4.742
		FY	1.838	1.490	0.611	-2.230	5.905
		MKT	0.913	1.490	0.927	-3.155	4.980
	MKT	Ctrl	-0.238	1.490	0.999	-4.305	3.830
		FY	0.925	1.490	0.924	-3.142	4.992
		GP	-0.913	1.490	0.927	-4.980	3.155
AST	Ctrl	FY	4.000	7.157	0.943	-15.542	23.542
		GP	-2.125	7.157	0.991	-21.667	17.417
		MKT	-4.750	7.157	0.910	-24.292	14.792
	FY	Ctrl	-4.000	7.157	0.943	-23.542	15.542
		GP	-6.125	7.157	0.827	-25.667	13.417
		MKT	-8.750	7.157	0.618	-28.292	10.792
	GP	Ctrl	2.125	7.157	0.991	-17.417	21.667
		FY	6.125	7.157	0.827	-13.417	25.667
		MKT	-2.625	7.157	0.983	-22.167	16.917
	MKT	Ctrl	4.750	7.157	0.910	-14.792	24.292
		FY	8.750	7.157	0.618	-10.792	28.292
		GP	2.625	7.157	0.983	-16.917	22.167
GGT	Ctrl	FY	1.875	1.987	0.782	-3.550	7.300
		GP	4.250	1.987	0.166	-1.175	9.675
		MKT	0.625	1.987	0.989	-4.800	6.050
	FY	Ctrl	-1.875	1.987	0.782	-7.300	3.550
		GP	2.375	1.987	0.635	-3.050	7.800
		MKT	-1.250	1.987	0.922	-6.675	4.175
	GP	Ctrl	-4.250	1.987	0.166	-9.675	1.175
		FY	-2.375	1.987	0.635	-7.800	3.050
		MKT	-3.625	1.987	0.284	-9.050	1.800
	MKT	Ctrl	-0.625	1.987	0.989	-6.050	4.800
		FY	1.250	1.987	0.922	-4.175	6.675
		GP	3.625	1.987	0.284	-1.800	9.050
Haptoglobin	Ctrl	FY	-0.054	0.047	0.664	-0.182	0.074
		GP	-0.005	0.047	1.000	-0.133	0.123
		MKT	-0.036	0.047	0.866	-0.164	0.092
	FY	Ctrl	0.054	0.047	0.664	-0.074	0.182
		GP	0.049	0.047	0.727	-0.079	0.177
		MKT	0.018	0.047	0.982	-0.110	0.145
	GP	Ctrl	0.005	0.047	1.000	-0.123	0.133
		FY	-0.049	0.047	0.727	-0.177	0.079
		MKT	-0.031	0.047	0.909	-0.159	0.097
	MKT	Ctrl	0.036	0.047	0.866	-0.092	0.164
		FY	-0.018	0.047	0.982	-0.145	0.110
		GP	0.031	0.047	0.909	-0.097	0.159
Insulin	Ctrl	FY	-0.096	0.245	0.979	-0.764	0.572
		GP	-0.286	0.245	0.650	-0.954	0.382
		MKT	-0.314	0.245	0.580	-0.982	0.354

	FY	Ctrl	0.096	0.245	0.979	-0.572	0.764
		GP	-0.190	0.245	0.864	-0.858	0.477
		MKT	-0.218	0.245	0.809	-0.886	0.449
	GP	Ctrl	0.286	0.245	0.650	-0.382	0.954
		FY	0.190	0.245	0.864	-0.477	0.858
		MKT	-0.028	0.245	0.999	-0.696	0.640
	MKT	Ctrl	0.314	0.245	0.580	-0.354	0.982
		FY	0.218	0.245	0.809	-0.449	0.886
		GP	0.028	0.245	0.999	-0.640	0.696
IGF-I	Ctrl	FY	18.529	46.016	0.977	-107.109	144.168
		GP	-17.817	46.016	0.980	-143.455	107.821
		MKT	-54.141	46.016	0.646	-179.779	71.497
	FY	Ctrl	-18.529	46.016	0.977	-144.168	107.109
		GP	-36.346	46.016	0.858	-161.984	89.292
		MKT	-72.671	46.016	0.406	-198.309	52.968
	GP	Ctrl	17.817	46.016	0.980	-107.821	143.455
		FY	36.346	46.016	0.858	-89.292	161.984
		MKT	-36.324	46.016	0.859	-161.962	89.314
	MKT	Ctrl	54.141	46.016	0.646	-71.497	179.779
		FY	72.671	46.016	0.406	-52.968	198.309
		GP	36.324	46.016	0.859	-89.314	161.962
Asn/Ser	Ctrl	FY	-11.279	24.759	0.968	-78.879	56.321
		GP	-17.528	24.759	0.893	-85.129	50.072
		MKT	-12.668	24.759	0.956	-80.268	54.932
	FY	Ctrl	11.279	24.759	0.968	-56.321	78.879
		GP	-6.249	24.759	0.994	-73.849	61.351
		MKT	-1.389	24.759	1.000	-68.989	66.211
	GP	Ctrl	17.528	24.759	0.893	-50.072	85.129
		FY	6.249	24.759	0.994	-61.351	73.849
		MKT	4.860	24.759	0.997	-62.740	72.461
	MKT	Ctrl	12.668	24.759	0.956	-54.932	80.268
		FY	1.389	24.759	1.000	-66.211	68.989
		GP	-4.860	24.759	0.997	-72.461	62.740
Glu	Ctrl	FY	12.634	8.519	0.461	-10.625	35.892
		GP	9.874	8.519	0.657	-13.385	33.132
		MKT	2.777	8.519	0.988	-20.481	26.035
	FY	Ctrl	-12.634	8.519	0.461	-35.892	10.625
		GP	-2.760	8.519	0.988	-26.019	20.498
		MKT	-9.857	8.519	0.658	-33.115	13.402
	GP	Ctrl	-9.874	8.519	0.657	-33.132	13.385
		FY	2.760	8.519	0.988	-20.498	26.019
		MKT	-7.097	8.519	0.838	-30.355	16.162
	MKT	Ctrl	-2.777	8.519	0.988	-26.035	20.481
		FY	9.857	8.519	0.658	-13.402	33.115
		GP	7.097	8.519	0.838	-16.162	30.355
Gly	Ctrl	FY	-13.827	31.406	0.971	-99.771	72.117
		GP	-45.272	31.406	0.485	-131.216	40.672

		MKT	-41.445	31.406	0.559	-127.389	44.499
	FY	Ctrl	13.827	31.406	0.971	-72.117	99.771
		GP	-31.446	30.341	0.730	-114.475	51.584
		MKT	-27.619	30.341	0.800	-110.649	55.411
	GP	Ctrl	45.272	31.406	0.485	-40.672	131.216
		FY	31.446	30.341	0.730	-51.584	114.475
		MKT	3.827	30.341	0.999	-79.203	86.856
	MKT	Ctrl	41.445	31.406	0.559	-44.499	127.389
		FY	27.619	30.341	0.800	-55.411	110.649
		GP	-3.827	30.341	0.999	-86.856	79.203
His/Gln	Ctrl	FY	1.315	50.898	1.000	-137.652	140.283
		GP	-16.956	50.898	0.987	-155.923	122.012
		MKT	-16.892	50.898	0.987	-155.860	122.075
	FY	Ctrl	-1.315	50.898	1.000	-140.283	137.652
		GP	-18.271	50.898	0.984	-157.238	120.696
		MKT	-18.207	50.898	0.984	-157.175	120.760
	GP	Ctrl	16.956	50.898	0.987	-122.012	155.923
		FY	18.271	50.898	0.984	-120.696	157.238
		MKT	0.064	50.898	1.000	-138.904	139.031
	MKT	Ctrl	16.892	50.898	0.987	-122.075	155.860
		FY	18.207	50.898	0.984	-120.760	157.175
		GP	-0.064	50.898	1.000	-139.031	138.904
Arg	Ctrl	FY	-13.023	29.495	0.971	-93.554	67.508
		GP	-40.439	29.495	0.527	-120.970	40.092
		MKT	-68.170	29.495	0.120	-148.702	12.361
	FY	Ctrl	13.023	29.495	0.971	-67.508	93.554
		GP	-27.416	29.495	0.789	-107.947	53.115
		MKT	-55.147	29.495	0.264	-135.678	25.384
	GP	Ctrl	40.439	29.495	0.527	-40.092	120.970
		FY	27.416	29.495	0.789	-53.115	107.947
		MKT	-27.731	29.495	0.784	-108.263	52.800
	MKT	Ctrl	68.170	29.495	0.120	-12.361	148.702
		FY	55.147	29.495	0.264	-25.384	135.678
		GP	27.731	29.495	0.784	-52.800	108.263
Thr	Ctrl	FY	0.602	32.337	1.000	-87.688	88.891
		GP	-5.682	32.337	0.998	-93.971	82.608
		MKT	.15.82824*	32.337	0.007	-204.118	-27.539
	FY	Ctrl	-0.602	32.337	1.000	-88.891	87.688
		GP	-6.283	32.337	0.997	-94.572	82.006
		MKT	.16.42975*	32.337	0.006	-204.719	-28.140
	GP	Ctrl	5.682	32.337	0.998	-82.608	93.971
		FY	6.283	32.337	0.997	-82.006	94.572
		MKT	.10.14663*	32.337	0.010	-198.436	-21.857
	MKT	Ctrl	.15.82824*	32.337	0.007	27.539	204.118
		FY	.16.42975*	32.337	0.006	28.140	204.719
		GP	.10.14663*	32.337	0.010	21.857	198.436

Ala	Ctrl	FY	6.074	29.525	0.997	-74.537	86.686
		GP	8.000	29.525	0.993	-72.611	88.612
		MKT	-22.706	29.525	0.868	-103.317	57.905
	FY	Ctrl	-6.074	29.525	0.997	-86.686	74.537
		GP	1.926	29.525	1.000	-78.685	82.537
		MKT	-28.781	29.525	0.765	-109.392	51.831
	GP	Ctrl	-8.000	29.525	0.993	-88.612	72.611
		FY	-1.926	29.525	1.000	-82.537	78.685
		MKT	-30.707	29.525	0.728	-111.318	49.905
	MKT	Ctrl	22.706	29.525	0.868	-57.905	103.317
		FY	28.781	29.525	0.765	-51.831	109.392
		GP	30.707	29.525	0.728	-49.905	111.318
Pro	Ctrl	FY	-3.856	22.452	0.998	-65.156	57.444
		GP	-26.240	22.452	0.651	-87.540	35.061
		MKT	-9.899	22.452	0.971	-71.199	51.401
	FY	Ctrl	3.856	22.452	0.998	-57.444	65.156
		GP	-22.384	22.452	0.752	-83.684	38.917
		MKT	-6.043	22.452	0.993	-67.343	55.257
	GP	Ctrl	26.240	22.452	0.651	-35.061	87.540
		FY	22.384	22.452	0.752	-38.917	83.684
		MKT	16.341	22.452	0.885	-44.960	77.641
	MKT	Ctrl	9.899	22.452	0.971	-51.401	71.199
		FY	6.043	22.452	0.993	-55.257	67.343
		GP	-16.341	22.452	0.885	-77.641	44.960
Cys	Ctrl	FY	0.047	8.542	1.000	-23.275	23.369
		GP	0.690	8.542	1.000	-22.632	24.012
		MKT	-1.411	8.542	0.998	-24.733	21.911
	FY	Ctrl	-0.047	8.542	1.000	-23.369	23.275
		GP	0.643	8.542	1.000	-22.679	23.965
		MKT	-1.458	8.542	0.998	-24.780	21.864
	GP	Ctrl	-0.690	8.542	1.000	-24.012	22.632
		FY	-0.643	8.542	1.000	-23.965	22.679
		MKT	-2.101	8.542	0.995	-25.423	21.221
	MKT	Ctrl	1.411	8.542	0.998	-21.911	24.733
		FY	1.458	8.542	0.998	-21.864	24.780
		GP	2.101	8.542	0.995	-21.221	25.423
Tyr	Ctrl	FY	-20.047	14.490	0.520	-59.610	19.517
		GP	-2.876	14.490	0.997	-42.440	36.687
		MKT	-0.430	14.490	1.000	-39.994	39.133
	FY	Ctrl	20.047	14.490	0.520	-19.517	59.610
		GP	17.170	14.490	0.641	-22.393	56.734
		MKT	19.617	14.490	0.538	-19.947	59.180
	GP	Ctrl	2.876	14.490	0.997	-36.687	42.440
		FY	-17.170	14.490	0.641	-56.734	22.393
		MKT	2.446	14.490	0.998	-37.117	42.010
	MKT	Ctrl	0.430	14.490	1.000	-39.133	39.994
		FY	-19.617	14.490	0.538	-59.180	19.947

		GP	-2.446	14.490	0.998	-42.010	37.117
Val	Ctrl	FY	8.587	34.655	0.995	-86.032	103.206
		GP	7.358	34.655	0.997	-87.261	101.977
		MKT	13.190	34.655	0.981	-81.429	107.809
	FY	Ctrl	-8.587	34.655	0.995	-103.206	86.032
		GP	-1.229	34.655	1.000	-95.848	93.390
		MKT	4.603	34.655	0.999	-90.016	99.222
	GP	Ctrl	-7.358	34.655	0.997	-101.977	87.261
		FY	1.229	34.655	1.000	-93.390	95.848
		MKT	5.832	34.655	0.998	-88.787	100.451
	MKT	Ctrl	-13.190	34.655	0.981	-107.809	81.429
		FY	-4.603	34.655	0.999	-99.222	90.016
		GP	-5.832	34.655	0.998	-100.451	88.787
Met	Ctrl	FY	0.263	8.942	1.000	-24.153	24.679
		GP	0.988	8.942	1.000	-23.428	25.403
		MKT	-37.22313*	8.942	0.001	-61.639	-12.807
	FY	Ctrl	-0.263	8.942	1.000	-24.679	24.153
		GP	0.724	8.942	1.000	-23.691	25.140
		MKT	-37.48630*	8.942	0.001	-61.902	-13.071
	GP	Ctrl	-0.988	8.942	1.000	-25.403	23.428
		FY	-0.724	8.942	1.000	-25.140	23.691
		MKT	-38.21068*	8.942	0.001	-62.626	-13.795
	MKT	Ctrl	37.22313*	8.942	0.001	12.807	61.639
		FY	37.48630*	8.942	0.001	13.071	61.902
		GP	38.21068*	8.942	0.001	13.795	62.626
Lys	Ctrl	FY	-20.507	42.963	0.963	-137.810	96.796
		GP	-11.095	42.963	0.994	-128.398	106.208
		MKT	-84.041	42.963	0.229	-201.343	33.262
	FY	Ctrl	20.507	42.963	0.963	-96.796	137.810
		GP	9.412	42.963	0.996	-107.891	126.715
		MKT	-63.533	42.963	0.463	-180.836	53.770
	GP	Ctrl	11.095	42.963	0.994	-106.208	128.398
		FY	-9.412	42.963	0.996	-126.715	107.891
		MKT	-72.946	42.963	0.344	-190.249	44.357
	MKT	Ctrl	84.041	42.963	0.229	-33.262	201.343
		FY	63.533	42.963	0.463	-53.770	180.836
		GP	72.946	42.963	0.344	-44.357	190.249
Ile	Ctrl	FY	2.347	16.993	0.999	-44.050	48.744
		GP	6.331	16.993	0.982	-40.066	52.728
		MKT	7.469	16.993	0.971	-38.928	53.866
	FY	Ctrl	-2.347	16.993	0.999	-48.744	44.050
		GP	3.984	16.993	0.995	-42.413	50.381
		MKT	5.123	16.993	0.990	-41.274	51.520
	GP	Ctrl	-6.331	16.993	0.982	-52.728	40.066
		FY	-3.984	16.993	0.995	-50.381	42.413
		MKT	1.138	16.993	1.000	-45.259	47.535

	MKT	Ctrl	-7.469	16.993	0.971	-53.866	38.928
		FY	-5.123	16.993	0.990	-51.520	41.274
		GP	-1.138	16.993	1.000	-47.535	45.259
Leu	Ctrl	FY	6.630	29.532	0.996	-74.002	87.262
		GP	13.832	29.532	0.965	-66.800	94.464
		MKT	23.157	29.532	0.861	-57.475	103.789
	FY	Ctrl	-6.630	29.532	0.996	-87.262	74.002
		GP	7.202	29.532	0.995	-73.430	87.834
		MKT	16.527	29.532	0.943	-64.104	97.159
	GP	Ctrl	-13.832	29.532	0.965	-94.464	66.800
		FY	-7.202	29.532	0.995	-87.834	73.430
		MKT	9.325	29.532	0.989	-71.306	89.957
	MKT	Ctrl	-23.157	29.532	0.861	-103.789	57.475
		FY	-16.527	29.532	0.943	-97.159	64.104
		GP	-9.325	29.532	0.989	-89.957	71.306
Phe	Ctrl	FY	-22.587	8.881	0.075	-46.834	1.661
		GP	-1.087	8.881	0.999	-25.334	23.160
		MKT	4.264	8.881	0.963	-19.983	28.511
	FY	Ctrl	22.587	8.881	0.075	-1.661	46.834
		GP	21.500	8.881	0.096	-2.748	45.747
		MKT	26.85050*	8.881	0.026	2.603	51.098
	GP	Ctrl	1.087	8.881	0.999	-23.160	25.334
		FY	-21.500	8.881	0.096	-45.747	2.748
		MKT	5.351	8.881	0.930	-18.896	29.598
	MKT	Ctrl	-4.264	8.881	0.963	-28.511	19.983
		FY	-26.85050*	8.881	0.026	-51.098	-2.603
		GP	-5.351	8.881	0.930	-29.598	18.896
Trp	Ctrl	FY	-2.468	8.346	0.991	-25.255	20.320
		GP	-6.313	8.346	0.873	-29.101	16.474
		MKT	-5.052	8.346	0.930	-27.840	17.735
	FY	Ctrl	2.468	8.346	0.991	-20.320	25.255
		GP	-3.846	8.346	0.967	-26.633	18.942
		MKT	-2.585	8.346	0.989	-25.372	20.203
	GP	Ctrl	6.313	8.346	0.873	-16.474	29.101
		FY	3.846	8.346	0.967	-18.942	26.633
		MKT	1.261	8.346	0.999	-21.527	24.049
	MKT	Ctrl	5.052	8.346	0.930	-17.735	27.840
		FY	2.585	8.346	0.989	-20.203	25.372
		GP	-1.261	8.346	0.999	-24.049	21.527

Data from dairy calves fed with control milk replacer (Ctrl); milk replacer supplemented with glycine and proline (GP); milk replacer supplemented with phenylalanine and tyrosine (FY) and milk replacer supplemented with lysine, methionine and threonine (MKT) (n=8 per group).

*: The mean difference is significant at the 0.05 level.

Table S4 Tukey HSD of ANOVA for quantified muscle metabolites from calves supplemented with different amino acids.

Dependent Variable			Mean Difference (I-J)	Std. Error	Sig.	95% Confidence Lower Bound	95% Confidence Upper Bound
3-Hydroxybutyrate	Ctrl	FY	-0.082	0.045	0.276	-0.205	0.040
		GP	-0.019	0.045	0.972	-0.142	0.103
		MKT	0.008	0.043	0.998	-0.111	0.126
	FY	Ctrl	0.082	0.045	0.276	-0.040	0.205
		GP	0.063	0.046	0.530	-0.063	0.190
		MKT	0.090	0.045	0.206	-0.032	0.213
	GP	Ctrl	0.019	0.045	0.972	-0.103	0.142
		FY	-0.063	0.046	0.530	-0.190	0.063
		MKT	0.027	0.045	0.929	-0.095	0.150
	MKT	Ctrl	-0.008	0.043	0.998	-0.126	0.111
		FY	-0.090	0.045	0.206	-0.213	0.032
		GP	-0.027	0.045	0.929	-0.150	0.095
Acetate	Ctrl	FY	-0.004	0.113	1.000	-0.313	0.305
		GP	0.035	0.113	0.990	-0.275	0.344
		MKT	0.059	0.109	0.948	-0.240	0.357
	FY	Ctrl	0.004	0.113	1.000	-0.305	0.313
		GP	0.039	0.116	0.987	-0.280	0.358
		MKT	0.063	0.113	0.943	-0.246	0.372
	GP	Ctrl	-0.035	0.113	0.990	-0.344	0.275
		FY	-0.039	0.116	0.987	-0.358	0.280
		MKT	0.024	0.113	0.996	-0.285	0.333
	MKT	Ctrl	-0.059	0.109	0.948	-0.357	0.240
		FY	-0.063	0.113	0.943	-0.372	0.246
		GP	-0.024	0.113	0.996	-0.333	0.285
ADP+ATP+AMP	Ctrl	FY	1.139	6.268	0.998	-16.055	18.333
		GP	-3.324	6.268	0.951	-20.518	13.870
		MKT	3.667	6.055	0.929	-12.944	20.278
	FY	Ctrl	-1.139	6.268	0.998	-18.333	16.055
		GP	-4.462	6.473	0.900	-22.220	13.296
		MKT	2.528	6.268	0.977	-14.666	19.722
	GP	Ctrl	3.324	6.268	0.951	-13.870	20.518
		FY	4.462	6.473	0.900	-13.296	22.220
		MKT	6.990	6.268	0.684	-10.204	24.184
	MKT	Ctrl	-3.667	6.055	0.929	-20.278	12.944
		FY	-2.528	6.268	0.977	-19.722	14.666
		GP	-6.990	6.268	0.684	-24.184	10.204
Alanine	Ctrl	FY	0.707	1.752	0.977	-4.100	5.514
		GP	0.428	1.752	0.995	-4.378	5.235
		MKT	0.118	1.693	1.000	-4.525	4.762
	FY	Ctrl	-0.707	1.752	0.977	-5.514	4.100
		GP	-0.279	1.810	0.999	-5.243	4.685

		MKT	-0.589	1.752	0.987	-5.395	4.218
	GP	Ctrl	-0.428	1.752	0.995	-5.235	4.378
		FY	0.279	1.810	0.999	-4.685	5.243
		MKT	-0.310	1.752	0.998	-5.116	4.497
	MKT	Ctrl	-0.118	1.693	1.000	-4.762	4.525
		FY	0.589	1.752	0.987	-4.218	5.395
		GP	0.310	1.752	0.998	-4.497	5.116
Anserine	Ctrl	FY	-1.455	3.099	0.965	-9.956	7.046
		GP	-3.357	3.099	0.702	-11.859	5.144
		MKT	0.374	2.994	0.999	-7.839	8.587
	FY	Ctrl	1.455	3.099	0.965	-7.046	9.956
		GP	-1.902	3.200	0.933	-10.682	6.878
		MKT	1.829	3.099	0.934	-6.672	10.330
	GP	Ctrl	3.357	3.099	0.702	-5.144	11.859
		FY	1.902	3.200	0.933	-6.878	10.682
		MKT	3.731	3.099	0.630	-4.770	12.233
	MKT	Ctrl	-0.374	2.994	0.999	-8.587	7.839
		FY	-1.829	3.099	0.934	-10.330	6.672
		GP	-3.731	3.099	0.630	-12.233	4.770
Aspartate	Ctrl	FY	-0.093	0.146	0.919	-0.494	0.308
		GP	0.117	0.146	0.854	-0.284	0.518
		MKT	-0.019	0.141	0.999	-0.406	0.368
	FY	Ctrl	0.093	0.146	0.919	-0.308	0.494
		GP	0.210	0.151	0.516	-0.204	0.624
		MKT	0.074	0.146	0.956	-0.327	0.475
	GP	Ctrl	-0.117	0.146	0.854	-0.518	0.284
		FY	-0.210	0.151	0.516	-0.624	0.204
		MKT	-0.136	0.146	0.790	-0.536	0.265
	MKT	Ctrl	0.019	0.141	0.999	-0.368	0.406
		FY	-0.074	0.146	0.956	-0.475	0.327
		GP	0.136	0.146	0.790	-0.265	0.536
Betaine	Ctrl	FY	-1.020	4.227	0.995	-12.616	10.576
		GP	-3.042	4.227	0.888	-14.638	8.554
		MKT	5.564	4.084	0.533	-5.638	16.767
	FY	Ctrl	1.020	4.227	0.995	-10.576	12.616
		GP	-2.022	4.366	0.966	-13.998	9.955
		MKT	6.584	4.227	0.419	-5.012	18.181
	GP	Ctrl	3.042	4.227	0.888	-8.554	14.638
		FY	2.022	4.366	0.966	-9.955	13.998
		MKT	8.606	4.227	0.201	-2.990	20.202
	MKT	Ctrl	-5.564	4.084	0.533	-16.767	5.638
		FY	-6.584	4.227	0.419	-18.181	5.012
		GP	-8.606	4.227	0.201	-20.202	2.990
Cadaverine	Ctrl	FY	1.043	0.613	0.343	-0.638	2.723
		GP	0.290	0.613	0.964	-1.391	1.970
		MKT	-1.088	0.592	0.279	-2.712	0.535
	FY	Ctrl	-1.043	0.613	0.343	-2.723	0.638

		GP	-0.753	0.633	0.638	-2.489	0.983
		MKT	07000000*	0.613	0.009	-3.812	-0.451
	GP	Ctrl	-0.290	0.613	0.964	-1.970	1.391
		FY	0.753	0.633	0.638	-0.983	2.489
		MKT	-1.378	0.613	0.137	-3.059	0.302
	MKT	Ctrl	1.088	0.592	0.279	-0.535	2.712
		FY	07000000*	0.613	0.009	0.451	3.812
		GP	1.378	0.613	0.137	-0.302	3.059
carnitine	Ctrl	FY	-2.901	3.098	0.786	-11.398	5.597
		GP	-5.231	3.098	0.350	-13.729	3.266
		MKT	2.674	2.993	0.808	-5.536	10.883
	FY	Ctrl	2.901	3.098	0.786	-5.597	11.398
		GP	-2.331	3.199	0.885	-11.107	6.445
		MKT	5.574	3.098	0.296	-2.923	14.072
	GP	Ctrl	5.231	3.098	0.350	-3.266	13.729
		FY	2.331	3.199	0.885	-6.445	11.107
		MKT	7.905	3.098	0.075	-0.592	16.403
	MKT	Ctrl	-2.674	2.993	0.808	-10.883	5.536
		FY	-5.574	3.098	0.296	-14.072	2.923
		GP	-7.905	3.098	0.075	-16.403	0.592
Carnosine	Ctrl	FY	-30.073	17.039	0.312	-76.817	16.670
		GP	-25.273	17.039	0.462	-72.017	21.470
		MKT	19.415	16.461	0.645	-25.743	64.574
	FY	Ctrl	30.073	17.039	0.312	-16.670	76.817
		GP	4.800	17.598	0.993	-43.477	53.076
		MKT	70000000*	17.039	0.035	2.745	96.232
	GP	Ctrl	25.273	17.039	0.462	-21.470	72.017
		FY	-4.800	17.598	0.993	-53.076	43.477
		MKT	44.689	17.039	0.065	-2.055	91.432
	MKT	Ctrl	-19.415	16.461	0.645	-64.574	25.743
		FY	70000000*	17.039	0.035	-96.232	-2.745
		GP	-44.689	17.039	0.065	-91.432	2.055
Creatine	Ctrl	FY	-11.394	38.432	0.991	-116.825	94.036
		GP	-35.597	38.432	0.791	-141.027	69.833
		MKT	27.574	37.128	0.879	-74.281	129.429
	FY	Ctrl	11.394	38.432	0.991	-94.036	116.825
		GP	-24.203	39.692	0.928	-133.091	84.685
		MKT	38.969	38.432	0.743	-66.462	144.399
	GP	Ctrl	35.597	38.432	0.791	-69.833	141.027
		FY	24.203	39.692	0.928	-84.685	133.091
		MKT	63.171	38.432	0.373	-42.259	168.601
	MKT	Ctrl	-27.574	37.128	0.879	-129.429	74.281
		FY	-38.969	38.432	0.743	-144.399	66.462
		GP	-63.171	38.432	0.373	-168.601	42.259
Creatine phosphate	Ctrl	FY	-9.517	23.794	0.978	-74.793	55.759
		GP	12.328	23.794	0.954	-52.947	77.604

		MKT	8.791	22.988	0.981	-54.271	71.854
	FY	Ctrl	9.517	23.794	0.978	-55.759	74.793
		GP	21.846	24.575	0.811	-45.571	89.262
		MKT	18.308	23.794	0.867	-46.967	83.584
	GP	Ctrl	-12.328	23.794	0.954	-77.604	52.947
		FY	-21.846	24.575	0.811	-89.262	45.571
		MKT	-3.537	23.794	0.999	-68.813	61.739
	MKT	Ctrl	-8.791	22.988	0.981	-71.854	54.271
		FY	-18.308	23.794	0.867	-83.584	46.967
		GP	3.537	23.794	0.999	-61.739	68.813
Creatinine	Ctrl	FY	-1.193	3.565	0.987	-10.971	8.586
		GP	1.276	3.565	0.984	-8.503	11.054
		MKT	-0.725	3.444	0.997	-10.172	8.722
	FY	Ctrl	1.193	3.565	0.987	-8.586	10.971
		GP	2.468	3.681	0.907	-7.631	12.568
		MKT	0.467	3.565	0.999	-9.311	10.246
	GP	Ctrl	-1.276	3.565	0.984	-11.054	8.503
		FY	-2.468	3.681	0.907	-12.568	7.631
		MKT	-2.001	3.565	0.943	-11.780	7.778
	MKT	Ctrl	0.725	3.444	0.997	-8.722	10.172
		FY	-0.467	3.565	0.999	-10.246	9.311
		GP	2.001	3.565	0.943	-7.778	11.780
Fumarate	Ctrl	FY	0.092	0.101	0.803	-0.187	0.370
		GP	-0.087	0.101	0.826	-0.365	0.191
		MKT	0.028	0.098	0.992	-0.241	0.297
	FY	Ctrl	-0.092	0.101	0.803	-0.370	0.187
		GP	-0.179	0.105	0.341	-0.466	0.109
		MKT	-0.064	0.101	0.921	-0.342	0.214
	GP	Ctrl	0.087	0.101	0.826	-0.191	0.365
		FY	0.179	0.105	0.341	-0.109	0.466
		MKT	0.115	0.101	0.674	-0.164	0.393
	MKT	Ctrl	-0.028	0.098	0.992	-0.297	0.241
		FY	0.064	0.101	0.921	-0.214	0.342
		GP	-0.115	0.101	0.674	-0.393	0.164
Glutamate	Ctrl	FY	-0.344	1.564	0.996	-4.635	3.948
		GP	-1.541	1.564	0.759	-5.832	2.751
		MKT	-0.123	1.511	1.000	-4.269	4.023
	FY	Ctrl	0.344	1.564	0.996	-3.948	4.635
		GP	-1.197	1.616	0.880	-5.629	3.235
		MKT	0.220	1.564	0.999	-4.071	4.512
	GP	Ctrl	1.541	1.564	0.759	-2.751	5.832
		FY	1.197	1.616	0.880	-3.235	5.629
		MKT	1.417	1.564	0.802	-2.874	5.709
	MKT	Ctrl	0.123	1.511	1.000	-4.023	4.269
		FY	-0.220	1.564	0.999	-4.512	4.071
		GP	-1.417	1.564	0.802	-5.709	2.874
Glycine	Ctrl	FY	1.951	4.766	0.976	-11.123	15.026

		GP	1.220	4.766	0.994	-11.854	14.295
		MKT	1.689	4.604	0.983	-10.942	14.320
	FY	Ctrl	-1.951	4.766	0.976	-15.026	11.123
		GP	-0.731	4.922	0.999	-14.234	12.772
		MKT	-0.262	4.766	1.000	-13.337	12.812
	GP	Ctrl	-1.220	4.766	0.994	-14.295	11.854
		FY	0.731	4.922	0.999	-12.772	14.234
		MKT	0.469	4.766	1.000	-12.605	13.543
	MKT	Ctrl	-1.689	4.604	0.983	-14.320	10.942
		FY	0.262	4.766	1.000	-12.812	13.337
		GP	-0.469	4.766	1.000	-13.543	12.605
Glucose	Ctrl	FY	1.860	3.073	0.929	-6.571	10.292
		GP	-1.693	3.073	0.946	-10.124	6.739
		MKT	1.809	2.969	0.928	-6.336	9.955
	FY	Ctrl	-1.860	3.073	0.929	-10.292	6.571
		GP	-3.553	3.174	0.681	-12.261	5.155
		MKT	-0.051	3.073	1.000	-8.482	8.381
	GP	Ctrl	1.693	3.073	0.946	-6.739	10.124
		FY	3.553	3.174	0.681	-5.155	12.261
		MKT	3.502	3.073	0.669	-4.929	11.934
	MKT	Ctrl	-1.809	2.969	0.928	-9.955	6.336
		FY	0.051	3.073	1.000	-8.381	8.482
		GP	-3.502	3.073	0.669	-11.934	4.929
Glucose-6-phosphate	Ctrl	FY	4.937	5.292	0.788	-9.580	19.454
		GP	-5.732	5.292	0.703	-20.249	8.785
		MKT	6.213	5.112	0.623	-7.811	20.238
	FY	Ctrl	-4.937	5.292	0.788	-19.454	9.580
		GP	-10.669	5.465	0.232	-25.662	4.324
		MKT	1.277	5.292	0.995	-13.240	15.794
	GP	Ctrl	5.732	5.292	0.703	-8.785	20.249
		FY	10.669	5.465	0.232	-4.324	25.662
		MKT	11.946	5.292	0.135	-2.571	26.463
	MKT	Ctrl	-6.213	5.112	0.623	-20.238	7.811
		FY	-1.277	5.292	0.995	-15.794	13.240
		GP	-11.946	5.292	0.135	-26.463	2.571
Isoleucine	Ctrl	FY	0.037	0.188	0.997	-0.478	0.551
		GP	0.244	0.188	0.572	-0.271	0.759
		MKT	0.196	0.181	0.703	-0.301	0.694
	FY	Ctrl	-0.037	0.188	0.997	-0.551	0.478
		GP	0.207	0.194	0.711	-0.325	0.739
		MKT	0.160	0.188	0.830	-0.355	0.674
	GP	Ctrl	-0.244	0.188	0.572	-0.759	0.271
		FY	-0.207	0.194	0.711	-0.739	0.325
		MKT	-0.048	0.188	0.994	-0.563	0.467
	MKT	Ctrl	-0.196	0.181	0.703	-0.694	0.301
		FY	-0.160	0.188	0.830	-0.674	0.355
		GP	0.048	0.188	0.994	-0.467	0.563

Lactate	Ctrl	FY	-4.704	71.813	1.000	-201.711	192.303
		GP	-96.682	71.813	0.543	-293.689	100.325
		MKT	34.455	69.378	0.959	-155.872	224.782
	FY	Ctrl	4.704	71.813	1.000	-192.303	201.711
		GP	-91.979	74.169	0.608	-295.447	111.489
		MKT	39.158	71.813	0.947	-157.849	236.165
	GP	Ctrl	96.682	71.813	0.543	-100.325	293.689
		FY	91.979	74.169	0.608	-111.489	295.447
		MKT	131.137	71.813	0.284	-65.870	328.144
	MKT	Ctrl	-34.455	69.378	0.959	-224.782	155.872
		FY	-39.158	71.813	0.947	-236.165	157.849
		GP	-131.137	71.813	0.284	-328.144	65.870
Leucine	Ctrl	FY	0.040	0.293	0.999	-0.764	0.845
		GP	0.236	0.293	0.852	-0.569	1.040
		MKT	0.266	0.283	0.785	-0.512	1.043
	FY	Ctrl	-0.040	0.293	0.999	-0.845	0.764
		GP	0.195	0.303	0.917	-0.636	1.026
		MKT	0.225	0.293	0.868	-0.579	1.030
	GP	Ctrl	-0.236	0.293	0.852	-1.040	0.569
		FY	-0.195	0.303	0.917	-1.026	0.636
		MKT	0.030	0.293	1.000	-0.774	0.835
	MKT	Ctrl	-0.266	0.283	0.785	-1.043	0.512
		FY	-0.225	0.293	0.868	-1.030	0.579
		GP	-0.030	0.293	1.000	-0.835	0.774
Mannose	Ctrl	FY	1.121	0.690	0.382	-0.771	3.013
		GP	-0.537	0.690	0.864	-2.429	1.355
		MKT	1.022	0.666	0.432	-0.806	2.850
	FY	Ctrl	-1.121	0.690	0.382	-3.013	0.771
		GP	-1.658	0.712	0.118	-3.612	0.296
		MKT	-0.099	0.690	0.999	-1.991	1.793
	GP	Ctrl	0.537	0.690	0.864	-1.355	2.429
		FY	1.658	0.712	0.118	-0.296	3.612
		MKT	1.559	0.690	0.134	-0.333	3.451
	MKT	Ctrl	-1.022	0.666	0.432	-2.850	0.806
		FY	0.099	0.690	0.999	-1.793	1.991
		GP	-1.559	0.690	0.134	-3.451	0.333
myo-inositol	Ctrl	FY	0.391	0.581	0.906	-1.202	1.985
		GP	-0.116	0.581	0.997	-1.710	1.477
		MKT	0.259	0.561	0.967	-1.281	1.798
	FY	Ctrl	-0.391	0.581	0.906	-1.985	1.202
		GP	-0.508	0.600	0.832	-2.154	1.138
		MKT	-0.133	0.581	0.996	-1.726	1.461
	GP	Ctrl	0.116	0.581	0.997	-1.477	1.710
		FY	0.508	0.600	0.832	-1.138	2.154
		MKT	0.375	0.581	0.916	-1.218	1.969
	MKT	Ctrl	-0.259	0.561	0.967	-1.798	1.281
		FY	0.133	0.581	0.996	-1.461	1.726

		GP	-0.375	0.581	0.916	-1.969	1.218
NAD+	Ctrl	FY	-0.419	0.310	0.540	-1.269	0.431
		GP	-0.250	0.310	0.851	-1.100	0.600
		MKT	0.694	0.299	0.120	-0.127	1.515
	FY	Ctrl	0.419	0.310	0.540	-0.431	1.269
		GP	0.169	0.320	0.952	-0.709	1.047
		MKT	42000000*	0.310	0.007	0.263	1.963
	GP	Ctrl	0.250	0.310	0.851	-0.600	1.100
		FY	-0.169	0.320	0.952	-1.047	0.709
		MKT	23000000*	0.310	0.025	0.094	1.794
	MKT	Ctrl	-0.694	0.299	0.120	-1.515	0.127
		FY	42000000*	0.310	0.007	-1.963	-0.263
		GP	23000000*	0.310	0.025	-1.794	-0.094
NADH	Ctrl	FY	0.178	0.235	0.873	-0.467	0.822
		GP	0.306	0.235	0.569	-0.339	0.951
		MKT	0.207	0.227	0.800	-0.416	0.829
	FY	Ctrl	-0.178	0.235	0.873	-0.822	0.467
		GP	0.129	0.243	0.951	-0.537	0.795
		MKT	0.029	0.235	0.999	-0.616	0.674
	GP	Ctrl	-0.306	0.235	0.569	-0.951	0.339
		FY	-0.129	0.243	0.951	-0.795	0.537
		MKT	-0.100	0.235	0.974	-0.744	0.545
	MKT	Ctrl	-0.207	0.227	0.800	-0.829	0.416
		FY	-0.029	0.235	0.999	-0.674	0.616
		GP	0.100	0.235	0.974	-0.545	0.744
NADP	Ctrl	FY	-0.005	0.014	0.988	-0.044	0.034
		GP	-0.013	0.014	0.805	-0.052	0.026
		MKT	-0.004	0.014	0.993	-0.041	0.034
	FY	Ctrl	0.005	0.014	0.988	-0.034	0.044
		GP	-0.008	0.015	0.943	-0.049	0.032
		MKT	0.001	0.014	1.000	-0.038	0.040
	GP	Ctrl	0.013	0.014	0.805	-0.026	0.052
		FY	0.008	0.015	0.943	-0.032	0.049
		MKT	0.009	0.014	0.917	-0.030	0.048
	MKT	Ctrl	0.004	0.014	0.993	-0.034	0.041
		FY	-0.001	0.014	1.000	-0.040	0.038
		GP	-0.009	0.014	0.917	-0.048	0.030
Nicotinurate	Ctrl	FY	-0.372	0.203	0.282	-0.929	0.185
		GP	94000000*	0.203	**0.014**	-1.230	-0.116
		MKT	0.016	0.196	1.000	-0.523	0.554
	FY	Ctrl	0.372	0.203	0.282	-0.185	0.929
		GP	-0.301	0.210	0.489	-0.877	0.274
		MKT	0.388	0.203	0.249	-0.170	0.945
	GP	Ctrl	94000000*	0.203	**0.014**	0.116	1.230
		FY	0.301	0.210	0.489	-0.274	0.877
		MKT	91000000*	0.203	**0.011**	0.132	1.246

	MKT	Ctrl	-0.016	0.196	1.000	-0.554	0.523
		FY	-0.388	0.203	0.249	-0.945	0.170
		GP	[illegible]91000000*	0.203	**0.011**	-1.246	-0.132
O-Acetylcarnitine	Ctrl	FY	0.208	1.183	0.998	-3.037	3.453
		GP	0.937	1.183	0.857	-2.308	4.182
		MKT	1.626	1.143	0.497	-1.509	4.760
	FY	Ctrl	-0.208	1.183	0.998	-3.453	3.037
		GP	0.729	1.222	0.932	-2.622	4.080
		MKT	1.418	1.183	0.633	-1.827	4.662
	GP	Ctrl	-0.937	1.183	0.857	-4.182	2.308
		FY	-0.729	1.222	0.932	-4.080	2.622
		MKT	0.688	1.183	0.937	-2.556	3.933
	MKT	Ctrl	-1.626	1.143	0.497	-4.760	1.509
		FY	-1.418	1.183	0.633	-4.662	1.827
		GP	-0.688	1.183	0.937	-3.933	2.556
Phenylalanine	Ctrl	FY	-0.103	0.072	0.496	-0.302	0.096
		GP	0.058	0.072	0.856	-0.141	0.256
		MKT	0.082	0.070	0.649	-0.110	0.274
	FY	Ctrl	0.103	0.072	0.496	-0.096	0.302
		GP	0.161	0.075	0.164	-0.044	0.366
		MKT	0.185	0.072	0.074	-0.013	0.384
	GP	Ctrl	-0.058	0.072	0.856	-0.256	0.141
		FY	-0.161	0.075	0.164	-0.366	0.044
		MKT	0.024	0.072	0.986	-0.174	0.223
	MKT	Ctrl	-0.082	0.070	0.649	-0.274	0.110
		FY	-0.185	0.072	0.074	-0.384	0.013
		GP	-0.024	0.072	0.986	-0.223	0.174
Pyruvate	Ctrl	FY	0.149	0.217	0.900	-0.446	0.744
		GP	-0.201	0.217	0.792	-0.795	0.394
		MKT	0.192	0.209	0.797	-0.383	0.766
	FY	Ctrl	-0.149	0.217	0.900	-0.744	0.446
		GP	-0.350	0.224	0.417	-0.964	0.264
		MKT	0.042	0.217	0.997	-0.552	0.637
	GP	Ctrl	0.201	0.217	0.792	-0.394	0.795
		FY	0.350	0.224	0.417	-0.264	0.964
		MKT	0.392	0.217	0.292	-0.203	0.987
	MKT	Ctrl	-0.192	0.209	0.797	-0.766	0.383
		FY	-0.042	0.217	0.997	-0.637	0.552
		GP	-0.392	0.217	0.292	-0.987	0.203
Taurine	Ctrl	FY	-3.166	2.325	0.534	-9.543	3.212
		GP	-2.077	2.325	0.808	-8.454	4.301
		MKT	1.289	2.246	0.939	-4.873	7.450
	FY	Ctrl	3.166	2.325	0.534	-3.212	9.543
		GP	1.089	2.401	0.968	-5.498	7.675
		MKT	4.454	2.325	0.246	-1.923	10.832
	GP	Ctrl	2.077	2.325	0.808	-4.301	8.454

		FY	-1.089	2.401	0.968	-7.675	5.498
		MKT	3.365	2.325	0.482	-3.012	9.743
	MKT	Ctrl	-1.289	2.246	0.939	-7.450	4.873
		FY	-4.454	2.325	0.246	-10.832	1.923
		GP	-3.365	2.325	0.482	-9.743	3.012
Tyrosine	Ctrl	FY	-0.171	0.111	0.435	-0.476	0.135
		GP	0.083	0.111	0.879	-0.223	0.389
		MKT	0.072	0.108	0.908	-0.223	0.368
	FY	Ctrl	0.171	0.111	0.435	-0.135	0.476
		GP	0.253	0.115	0.150	-0.063	0.569
		MKT	0.243	0.111	0.157	-0.063	0.548
	GP	Ctrl	-0.083	0.111	0.879	-0.389	0.223
		FY	-0.253	0.115	0.150	-0.569	0.063
		MKT	-0.011	0.111	1.000	-0.317	0.295
	MKT	Ctrl	-0.072	0.108	0.908	-0.368	0.223
		FY	-0.243	0.111	0.157	-0.548	0.063
		GP	0.011	0.111	1.000	-0.295	0.317
Tryptophan	Ctrl	FY	0.024	0.025	0.769	-0.044	0.091
		GP	-0.002	0.025	1.000	-0.070	0.065
		MKT	0.005	0.024	0.997	-0.061	0.070
	FY	Ctrl	-0.024	0.025	0.769	-0.091	0.044
		GP	-0.026	0.025	0.732	-0.096	0.044
		MKT	-0.019	0.025	0.863	-0.087	0.048
	GP	Ctrl	0.002	0.025	1.000	-0.065	0.070
		FY	0.026	0.025	0.732	-0.044	0.096
		MKT	0.007	0.025	0.991	-0.060	0.075
	MKT	Ctrl	-0.005	0.024	0.997	-0.070	0.061
		FY	0.019	0.025	0.863	-0.048	0.087
		GP	-0.007	0.025	0.991	-0.075	0.060
Valine	Ctrl	FY	0.202	0.356	0.941	-0.775	1.179
		GP	0.391	0.356	0.695	-0.587	1.368
		MKT	0.526	0.344	0.436	-0.418	1.470
	FY	Ctrl	-0.202	0.356	0.941	-1.179	0.775
		GP	0.189	0.368	0.955	-0.820	1.198
		MKT	0.324	0.356	0.800	-0.653	1.301
	GP	Ctrl	-0.391	0.356	0.695	-1.368	0.587
		FY	-0.189	0.368	0.955	-1.198	0.820
		MKT	0.135	0.356	0.981	-0.842	1.112
	MKT	Ctrl	-0.526	0.344	0.436	-1.470	0.418
		FY	-0.324	0.356	0.800	-1.301	0.653
		GP	-0.135	0.356	0.981	-1.112	0.842

Data from dairy calves fed with control milk replacer (Ctrl, n=8); milk replacer supplemented with glycine and proline (GP, n=7); milk replacer supplemented with phenylalanine and tyrosine (FY, n=7) and milk replacer supplemented with lysine, methionine and threonine (MKT, n=8).

*: The mean difference is significant at the 0.05 level.
